# Spatial proximity to national 5A tourist attractions and corporate environmental governance: Evidence from China

**DOI:** 10.1371/journal.pone.0345770

**Published:** 2026-03-26

**Authors:** Xiancheng Xiong, Chen Zhong

**Affiliations:** School of Economics, Management and Law, Jiangxi Science and Technology Normal University, Nanchang, China; Pegaso Telematic University: Universita Telematica Pegaso, ITALY

## Abstract

Tourism development is increasingly linked to both regional economic activity and environmental outcomes. However, empirical evidence remains limited on whether and through which channels proximity to major tourist attractions is associated with the environmental governance of industrial enterprises. Using enterprise-level data and geocoded locations of China’s national 5A tourist attractions in 2014, we examine the relationship between firms’ spatial proximity to 5A attractions and corporate environmental governance. We find that (1) proximity to 5A attractions is positively related to the environmental governance of nearby firms, and the results are robust across alternative specifications and a range of robustness checks, including IV-based estimates; (2) proximity is associated with lower operating costs, consistent with shared transportation infrastructure and environmental facilities, and with higher fixed-asset intensity that is linked to stronger environmental governance; and (3) the positive relationship is more pronounced for natural-landscape attractions and less pronounced in cities with higher innovation or marketization. These findings provide evidence consistent with positive environmental spillovers associated with tourism and underscore the potential value of coordinating tourism development strategies with industrial environmental governance policies.

## 1. Introduction

Currently, tourism is broadly seen as a key impetus for economic growth in many countries [1]. Tourism refers to travel outside one’s usual environment for business, leisure, or other personal purposes, typically for less than one year, without being employed by resident entities in the visited place [2]. A number of studies have demonstrated the critical role of tourism in capital accumulation [3], poverty alleviation [4], and social welfare improvement [5]. These direct effects of tourism typically involve changes in “sales [6], employment [7], tax revenue [8], and income levels [9]” and are widely observed across tourism-related industries. Beyond its economic role, tourism is also linked to environmental outcomes [10]. Prior research reports mixed evidence, documenting both environmental pressures (e.g., emissions, resource use, waste, and pollution) and potential environmental benefits under certain conditions [11–13].

Tourism is widely recognized to impose substantial environmental pressures through higher carbon emissions, natural resource overuse, and increased solid waste and water pollution [14–16]. At the same time, evidence on tourism’s potential environmental benefits remains mixed and context-dependent. Some studies suggest that tourism can raise public awareness and strengthen policy attention to natural resource protection, encouraging local governments to adopt environmental measures [17,18]. Others find that tourism development may stimulate local investment in clean energy, waste management, and environmental infrastructure [19–21]. Finally, a strand of research proposes a non-linear pattern consistent with a tourism-environment Kuznets-type relationship, in which environmental quality may initially deteriorate but later improve as awareness and technology advance [22].

A growing firm-level literature suggests that environmental regulation reshapes corporate behavior, both by increasing compliance costs and, under some policy designs, by encouraging cleaner technologies and green innovation [23–25]. Beyond within-firm responses, recent evidence points to spatial interdependence in regulation and environmental governance. Firms can respond on location margins, including entry, exit, and relocation. Moreover, policy effects may spill over to nearby jurisdictions through market linkages and strategic interactions [26–30]. In parallel, work on place-based policies shows that geographically targeted interventions can shape local firm entry, capital accumulation, productivity, and agglomeration, highlighting that location is an important determinant of firm-level outcomes [31–34].

However, these effects are heterogeneous. Program design and institutional features can determine whether zones raise productivity or instead generate distortions and redistribution within targeted areas. More broadly, a growing literature emphasizes that place-based interventions may involve equity-efficiency tradeoffs and displacement risks. This makes it important to understand the mechanisms through which localized advantages translate into firm-level outcomes. Taken together, this literature suggests that firms’ exposure to localized amenities and policies increases with geographic proximity. Yet relatively little is known about whether and through which channels tourism-oriented place-based development, such as proximity to national 5A tourist attractions, is associated with firm-level environmental governance.

Corporate environmental governance has increasingly been studied as a firm-level response to local institutions, infrastructure, and place-based conditions. Yet national 5A tourist attractions have rarely been examined as spatial factors that may shape firms’ environmental governance through local spillovers. Building on this gap, our study examines how firms’ spatial proximity to these attractions is associated with corporate environmental governance. We provide suggestive evidence on potential channels through which tourism-related local conditions may be linked to firms’ environmental governance.

To collect evidence, this study is focused on China, which is a developing country that actively fosters its tourism industry. In recent years, China’s tourism sector has demonstrated notable achievements and has garnered substantial attention from the central government [35]. For this study, we employ a cross-sectional survey dataset taken from the Chinese Industrial Enterprise Database and national 5A Tourist Attractions during the year of 2014 to construct our empirical model, and we do this for two reasons. First, by 2014, China had established a large number of national 5A attractions across the country. These attractions are the highest-rated tourist destinations in China and span natural landscapes, historical monuments, and cultural sites.

We use 2014 partly because it predates the tightening of China’s environmental enforcement in 2015. This helps keep the regulatory environment relatively stable in our cross-sectional analysis. In particular, the revised Environmental Protection Law took effect in 2015 and signaled a shift toward stricter enforcement aimed at reducing pollution and improving environmental quality. Second, the China Industrial Enterprises Database is a survey-based dataset covering a broad set of industrial firms nationwide, which provides a large cross-sectional sample. We additionally report IV-based estimates as a supplementary analysis to assess the sensitivity of our results to endogeneity concerns. Because we use a cross section, the exclusion restriction cannot be fully verified.

The contributions of this paper include the following: First, we bridge the tourism-environment and firm-level environmental governance literatures by providing firm-level evidence on how proximity to major tourist attractions relates to corporate environmental governance. Existing tourism studies typically focus on destination-, city-, or regional-level environmental indicators [36–38], while firm-level research rarely considers tourism-related local development as a spatial determinant of environmental governance. We link enterprise microdata with geocoded national 5A attractions to examine firms’ responses to attraction-induced local conditions.

Second, we show that the estimated relationship is robust across alternative specifications and a set of endogeneity-related checks, including IV-based analysis. Third, we offer suggestive evidence on mechanisms and heterogeneity: proximity to 5A attractions is associated with lower operating costs, potentially reflecting shared transport infrastructure and environmental facilities, and with higher fixed-asset intensity that is linked to stronger environmental governance. More broadly, our results suggest that national tourist attractions, together with the public infrastructure and environmental services that accompany them, may affect nearby firms’ environmental governance beyond China.

The remainder of this paper is organized as follows. Section 2 develops the hypotheses. Section 3 describes the data and empirical strategy. Section 4 presents the results. Section 5 explores mechanisms and additional robustness checks. Section 6 concludes.

## 2. Theoretical analysis and hypothesis development

### 2.1. Proximity to 5A tourist attractions, shared infrastructure, and firms’ operating costs

The theory of the geographical advantage of tourism, which is a core concept in tourism geography and regional economics, explains the spatial distribution of tourism resources, the formation of tourist destinations, and the locational choices that support tourism industry development [39]. The theory proposes that tourist attractions exert significant economic spillovers on their surrounding areas [40,41]. China classifies tourist attractions into five quality tiers, from A to AAAAA, under a centralized evaluation system. National 5A attractions represent the highest tier and are designated by the central government based on strict criteria. One key requirement is accessibility. In practice, 5A attractions are expected to be well connected by major transport modes such as air travel, high-speed rail, expressways, or high-standard waterways. The designation also imposes minimum thresholds for scale and visitation. Each attraction must cover at least three square kilometres and receive at least 600,000 visitors annually, including at least 50,000 overseas visitors. The attraction must have been upgraded from 4A after having been maintained at 4A for at least three consecutive years [42].

Therefore, 5A tourist attractions have a significant spillover effect on nearby industrial enterprises. The planning and construction of 5A-level tourist attractions are often accompanied by substantial local government investments in the regional infrastructure, including upgrades to critical environmental facilities such as transportation networks, water and electricity supply, and wastewater treatment systems. These infrastructure expansions not only support the tourism industry but also create favourable external conditions for nearby non-tourism businesses. For instance, industrial enterprises can comply with discharge standards through the use of centralized wastewater treatment systems, thereby avoiding the high costs associated with the construction and operation of individual facilities [43,44]. Such a shared infrastructure significantly reduces the production, transportation, and environmental governance costs for businesses, particularly for industrial enterprises. Consequently, the infrastructure improvements associated with tourist attractions serve as an important mechanism for lowering operational costs for nearby industrial firms. In summary, we hypothesize that proximity to 5A tourist attractions may be linked to lower operating costs for nearby industrial enterprises.

### 2.2. Proximity to 5A tourist attractions and firms’ environmental governance strategies

We hypothesize that proximity to 5A tourist attractions is associated with stronger environmental governance among nearby firms, and we explore two candidate mechanisms. First, the development and operation of such attractions not only highlight the overall appeal of the regional tourism industry but are also closely tied to local government performance evaluations and economic development objectives [45]. To safeguard the stability and sustainability of the surrounding ecological environment, local environmental authorities often impose stricter and more frequent regulations on nearby enterprises to mitigate the adverse effects of pollution on tourism brands. This differentiated regulatory approach creates a form of “selective enforcement,” making those industrial enterprises located in proximity to scenic areas more likely targets of environmental law enforcement [46]. Under the combined influence of governmental “window guidance” and the pressure of public opinion, firms tend to proactively improve their environmental governance as a means of avoiding regulatory risks and reputational damage, even in the absence of strong intrinsic motivation [47]. Consequently, we hypothesize that tourist attractions are associated with spillovers in environmental regulation, which may provide an external incentive for nearby non-tourism firms to improve their environmental performance.

Second, the construction and operation of 5A-level tourist attractions result in a continuous influx of visitors, which substantially increases the levels of public attention and media exposure in the region, thereby elevating societal expectations regarding the quality of the surrounding ecological environment. In this highly visible context, pollution problems are more likely to attract public scrutiny and complaints, particularly as monitoring by local residents, tourists, and environmental organizations is becoming more frequent. This heightened public oversight constitutes an “informal institutional constraint” that generates persistent reputational pressure and social accountability risks for nearby businesses [48]. To maintain legitimacy and avoid reputational damage, firms often proactively strengthen their environmental governance and adopt more standardized environmental practices to procure the “social licence” necessary for sustained operations [49]. Thus, tourist attractions can shape the local social environment, which may affect not only tourism-related industries but also the environmental governance of nearby industrial enterprises through social pressure.

However, environmental governance is inherently capital intensive [50–52]. Whether it involves the purchase of wastewater treatment equipment or the adoption of clean production processes, firms need to strengthen their environmental governance capacities through substantial fixed asset investment. An increase in the fixed asset ratio, particularly for environmental protection-related assets, therefore serves as a tangible indicator of improved corporate environmental governance. From the perspective of corporate behavioural drivers, Porter’s hypothesis and its extended theoretical framework suggest that, rather than undermining competitiveness, moderate environmental regulation can stimulate technological upgrades in production and governance through “incentive–innovation” mechanisms [53,54]. In such cases, firms may respond by increasing capital investment in environmental governance to meet compliance requirements or protect reputation, which may improve their environmental performance.

Building on this logic, we hypothesize that proximity to 5A tourist attractions is associated with lower operating costs for nearby industrial enterprises. Lower operating costs may relax constraints and be linked to higher fixed-asset investment, including investment in environmental equipment, which is in turn associated with stronger environmental governance. Based on this reasoning, we propose the following hypotheses.

**Hypothesis 1:** Proximity to 5A tourist attractions is positively related to corporate environmental governance among nearby industrial enterprises.

**Hypothesis 2:** Proximity to 5A tourist attractions is negatively related to operating costs and positively related to fixed-asset intensity.

**Hypothesis 3:** Fixed-asset intensity is positively related to corporate environmental governance, and it partially accounts for the relationship between proximity to 5A tourist attractions and corporate environmental governance.

### 2.3. Heterogeneity analysis

We suggest that the nature of 5A tourist attractions (e.g., natural vs. cultural landscapes) and the differing levels of technological innovation and business environments in their host cities may condition how proximity to 5A tourist attractions relates to firms’ environmental governance. Accordingly, we examine whether attraction characteristics and local institutional environments are associated with heterogeneity in the relationship between proximity to 5A tourist attractions and firms’ environmental governance.

#### 2.3.1. Nature of 5A tourist attractions.

China’s 5A tourist attractions fall into two categories: natural and cultural landscapes. Natural landscapes form around natural resources, such as the Jade Dragon Snow Mountain in Yunnan Province, and are often located far from urban centres. Attractions located farther from central cities may be less exposed to urban innovation ecosystems and high-quality business environments. This may limit nearby firms’ access to frontier technologies and constrain their ability to upgrade environmental governance through technology-intensive approaches.

In contrast, cultural landscapes emerge from human activities and civilization, and they feature historical or artistic traits (e.g., the Forbidden City Museum and the Old Summer Palace). Typically situated in central cities related to specific historical events, these sites expose the surrounding industrial enterprises to advanced transportation systems as well as to higher levels of technological innovation and business environments. Consequently, higher levels of urban innovation or marketization may relax technological and institutional constraints faced by nearby firms. This may strengthen their ability to adopt cleaner production upgrades and improve environmental governance. We thus propose the following hypothesis:

**Hypothesis 4:** The positive relationship between proximity to 5A tourist attractions and firms’ environmental governance is more pronounced when the attraction is a natural landscape.

#### 2.3.2. City innovation ability.

To further motivate Hypothesis 4, we consider how the host city’s innovation environment may shape firms’ environmental governance responses. When local innovation capacity is higher, firms may face fewer constraints in upgrading environmental governance through technology-intensive and cleaner production approaches, partly by adopting and adapting existing technologies rather than undertaking frontier innovation. Relative to original innovation, technology adoption and imitation can reduce adjustment costs and implementation risks in environmental management [55]. Such upgrades may deliver both environmental improvements and private returns by improving efficiency and reducing regulatory and reputational risks. Based on this reasoning, we propose the following supplementary hypothesis:

**Hypothesis 4.1:** The positive relationship between proximity to 5A tourist attractions and firms’ environmental governance is less pronounced in cities with higher levels of innovation.

#### 2.3.3. City marketization.

We also collect city-level measures of marketization to further examine Hypothesis 4. In cities with higher marketization, credit allocation tends to rely more on market-based mechanisms, which may reduce firms’ incentives to engage in non-market activities to obtain financing [56,57]. A more market-oriented environment may also facilitate technology adoption and cleaner production upgrades by improving access to finance, inputs, and competitive pressure. As a result, the relationship between proximity to 5A tourist attractions and firms’ environmental governance may differ across cities with different marketization levels. Based on this reasoning, we propose the following hypothesis.

**Hypothesis 4.2**: The positive relationship between proximity to 5A tourist attractions and firms’ environmental governance is less pronounced in cities with higher marketization.

**Fig 1:**
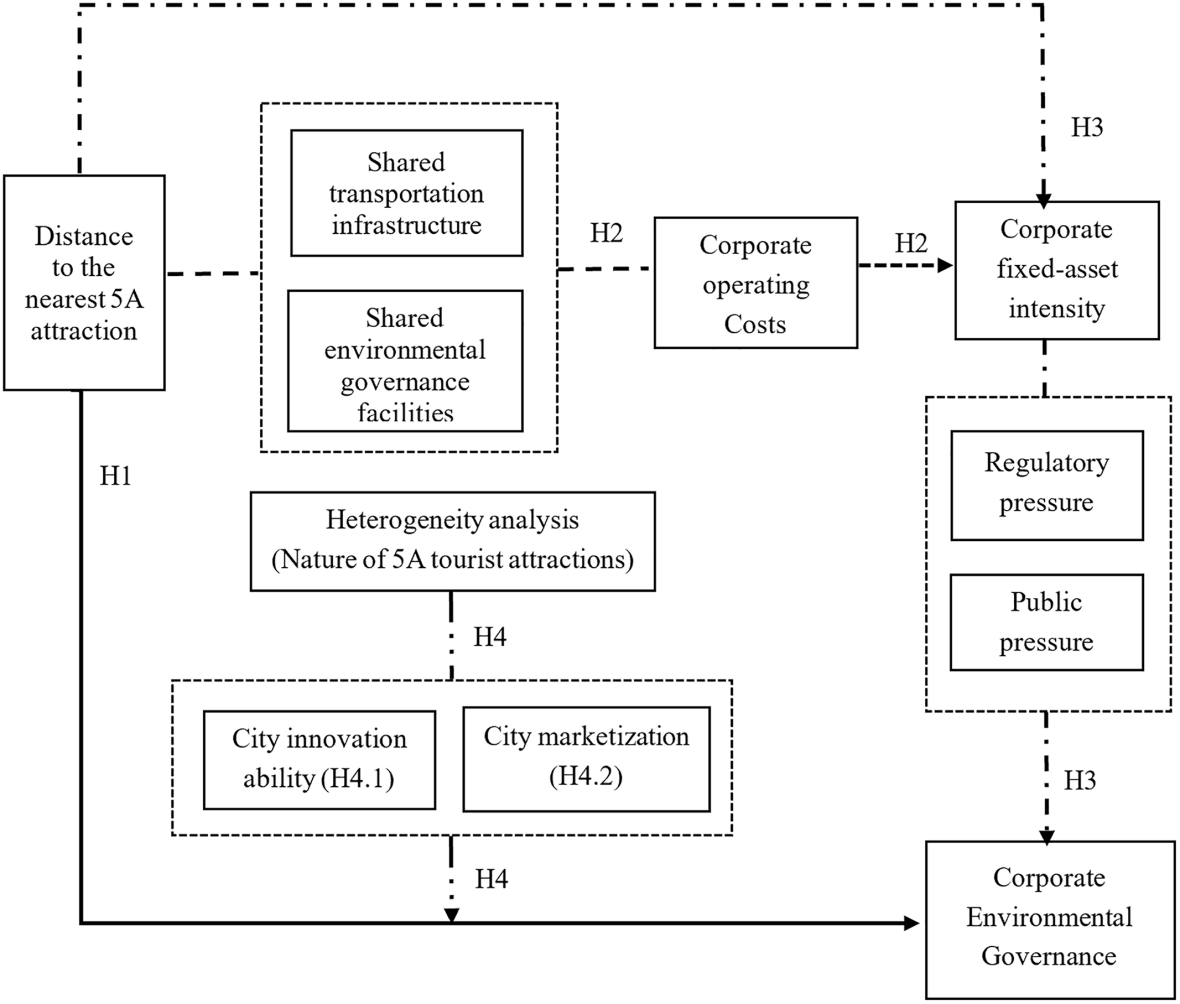
Framework of the Theoretical Analysis.

The theoretical analysis framework used in this study is presented in Fig 1.

## 3. Sample data and empirical identification

### 3.1. Data

#### 3.1.1. Sample.

This study did not involve human participants or animal subjects. Ethical approval and informed consent were not required as the research used publicly available corporate data. To examine the relationship between firms’ spatial proximity to China’s national 5A tourist attractions and corporate environmental governance, we use the 2014 survey wave of the China Industrial Enterprises Database. This database covers the industrial enterprises in 31 provinces across mainland China, with each reporting sales of above 5 million yuan. The database includes various enterprise types, such as state-owned, privately owned, and foreign-invested firms. We also retrieve data on the total volume of wastewater treatment from the database and then match it to the sampled enterprises. Additionally, we clean the dataset by removing anomalies, including any enterprises with zero registered capital and those reporting an average annual workforce of zero.

### 3.2. Main variables

#### 3.2.1. Shortest geographical distance from an industrial enterprise to a 5A tourist attraction.

To construct our key variable of interest, firms’ proximity to 5A tourist attractions, we proceed as follows: First, we gather the latitude and longitude of all 5A tourist attractions in China during the years of 2014 and 2023, along with the geographical coordinates of each industrial enterprise. Next, we use R software to calculate the geographical distance between each industrial enterprise and the nearest 5A tourist attraction. Finally, we measure a firm’s proximity to 5A tourist attractions using the shortest distance from the firm to the nearest 5A attraction **(Dis2014, Dis2023**). This approach follows the related literature, which suggests that the influence of tourist amenities on nearby firms and local conditions attenuates with distance [58]. Therefore, we treat firms located closer to 5A tourist attractions as having greater exposure to tourism-related local conditions. The geographic coordinates of China’s 5A attractions are obtained from the official website of the Ministry of Culture and Tourism.

#### 3.2.2. Environmental governance of enterprises.

We measure enterprises’ environmental governance (**CEG**) through the industrial wastewater treatment rate, which is calculated as the total treated industrial wastewater divided by the firm’s total fixed assets. This indicator is especially important because wastewater, compared with other pollutants, is more easily detected by both environmental regulators and non-governmental organizations. Therefore, the wastewater treatment rate offers a more accurate reflection of the environmental governance of industrial enterprises (industrial enterprise wastewater treatment data are taken from the Chinese Ministry of Ecology and Environment, and they reflect information on environmental pollution and governance, industrial enterprise emissions and environmental management).

#### 3.2.3. Control variables.

Drawing on the literature, we adopt several control variables [59,60]. We define **Size** as the natural log of the average annual number of employees, **lnAge** as the natural log of the enterprise’s years in operation and **Debt** as the natural log of total long-term liabilities. **Nature** is a dummy variable indicating the enterprise’s ownership type, and it takes a value of 1 if state-owned capital accounts for at least 50% and 0 otherwise. The enterprise’s ownership type is a critical influencing factor on its environmental governance. The literature indicates that in China, state-owned enterprises typically exhibit stronger environmental governance than private enterprises do, owing to more stringent environmental regulations. **Subsidy** is the enterprise’s subsidy income rate, which is calculated by dividing its annual subsidy income by its total paid-in capital. **Lighting** represents the nighttime light intensity in the enterprise’s city, which indirectly reflects human activity and serves as a proxy for the city’s economic development level [61]. **Pline** indicates whether the enterprise is located in a county that borders a provincial boundary, and it is based on the enterprise’s geographical coordinates. Our assumption is that those enterprises located closer to provincial boundaries typically operate under less developed economic conditions. **Coast** identifies whether an enterprise is situated in a coastal county (based on latitude and longitude), and it indicates an advantage in import-export trade (nighttime light data are obtained from the DMSP-OLS dataset provided by NOAA’s National Geophysical Data Center (https://ngdc.noaa.gov/eog/dmsp/downloadV4composites.html)).

Table 1 shows the input-output indicators. A correlation coefficient test was conducted on all the variables listed inTable 2, revealing significant correlations among most of them.

**Table 1:** Descriptive statistics.

Variable	Obs	Mean	Std.Dev.	Min	Max
CEG	18411	0.181	0.749	0	5.938
Dis2014	303717	10.498	0.993	3.811	15.636
Dis2023	303717	10.292	0.939	3.811	15.611
Control variables					
Size	291271	5.505	1.119	1.099	12.372
lnAge	292544	2.089	0.803	0	7.608
Debt	167705	8.175	2.389	0	19.588
Nature	303717	0.259	0.438	0	1
Subsidy	276540	9.423	1.986	0	19.144
Lighting	301871	20.124	16.582	0.001	55.793
Pline	301872	0.222	0.416	0	1
Coast	301872	0.247	0.431	0	1

**Table 2:** Correlation matrix.

	CEG	Dis2014	Dis2023	Size	lnAge	Debt	Nature	Subsidy	Lighting	Pline	Coast
CEG	1										
Dis2014	−0.04***	1									
Dis2023	−0.04***	0.86***	1								
Size	−0.15***	0.04***	0.04***	1							
lnAge	−0.03***	−0.11***	−0.11***	0.16***	1						
Debt	−0.13***	0.07***	0.06***	0.37***	0.09***	1					
Nature	−0.03***	−0.15***	−0.12***	0.18***	0.10***	0.05***	1				
Subsidy	−0.33***	0.15***	0.13***	0.45***	0.12***	0.65***	0.05***	1			
Lighting	0.05***	−0.53***	−0.43***	−0.04***	0.05***	−0.12***	0.24***	−0.21***	1		
Pline	0.00	0.08***	0.11***	−0.01***	−0.04***	0.01**	−0.06***	0.04***	−0.04***	1	
Coast	0.03***	−0.05***	−0.03***	0.01***	0.00	−0.05***	0.18***	−0.10***	0.35***	−0.21***	1

***, **, and * represent statistical significance at the 1%, 5%, and 10% level.

### 3.3. Empirical identifications

#### 3.3.1. Benchmark model.

To examine how proximity to 5A tourist attractions is associated with enterprises’ environmental governance, we employ the following empirical model:


$${\text{CEG}}_{\text{i},\text{p}}=\alpha_{\text{0}}+\alpha_{\text{1}}{\text{Dis2014}}_{\text{i},\text{p}}+\sum \alpha_{\text{i}}{\text{Control}}_{\text{i},\text{p}}+\text{prefecture fixed effects}+\epsilon_{\text{i},\text{p}}$$ (1)

where $\alpha_{\text{1}}$ is the coefficient of interest; i and p denote the firm and prefecture, respectively; ${Control}_{i,p}$ is a vector of the control variables; and $\varepsilon_{i,p}$ is the error term. On the basis of our theoretical framework, we expect $\alpha_{\text{1}}\;$<0, implying that a shorter distance (i.e., greater proximity) to the nearest 5A tourist attraction is associated with stronger environmental governance among nearby firms.

#### 3.3.2. Potential mechanisms linking proximity to 5A tourist attractions and corporate environmental governance.

Considering the proposed mechanisms, we examine whether operating costs and fixed-asset intensity are potential channels linking firms’ proximity to 5A tourist attractions and corporate environmental governance.


$${\text{Mediator}}_{\text{i},\text{p}}=\beta_{\text{0}}+\beta_{\text{1}}{\text{Dis2014}}_{\text{i},\text{p}}+\sum \beta_{\text{i}}{\text{Control}}_{\text{i},\text{p}}+\text{prefecture fixed effects}+\epsilon_{\text{i},\text{p}}$$ (2)

$${\text{CEG}}_{\text{i},\text{p}}=\overset{\prime }{\underset{\text{0}}{\alpha }}+\overset{\prime }{\underset{\text{1}}{\alpha }}{\text{Dis2014}}_{\text{i},\text{p}}+\overset{\prime }{\underset{\text{2}}{\alpha }}{\text{Mediator}}_{\text{i},\text{p}}+\sum \overset{\prime }{\underset{\text{i}}{\alpha }}{\text{Control}}_{\text{i},\text{p}}+\text{prefecture fixed effects}+\epsilon_{\text{i},\text{p}}$$ (3)

In Equation (3), we explore a mediation-type relationship in which proximity to 5A tourist attractions is associated with operating costs and fixed-asset intensity, which may help account for the association with corporate environmental governance. The cost rate (**Cost**) is defined as business operating costs divided by total paid-in capital, and fixed-asset intensity (**CFI**) is defined as total fixed assets divided by total paid-in capital.

To test for heterogeneity, we define three dummy variables as follows: 𝐋𝐚𝐧𝐝𝐬 = 1 if the 5A tourist attraction is natural and 0 otherwise (see **Section 5.4.1**); 𝐈𝐧𝐧𝐨𝐯𝐚𝐭𝐢𝐨𝐧 = 1  if the enterprise’s city has a high level of innovation and 0 otherwise (see **Section 5.4.2**); and 𝐌𝐚𝐫𝐤𝐞𝐭 =1  if the city has a high marketization level and 0 otherwise (see **Section 5.4.3**).


$$\begin{array}{cc} & {\text{CEG}}_{\text{i},\text{p}}=\gamma_{\text{0}}+\gamma_{\text{1}}{\text{Dis2014}}_{\text{i},\text{p}}+\gamma_{\text{2}}{\text{Mediator}}_{\text{i},\text{p}}+\gamma_{\text{3}}{\text{Mediator}}_{\text{i},\text{p}}\times {\text{Dis2014}}_{\text{i},\text{p}}+\\ & \sum \gamma_{\text{4}}{\text{Control}}_{\text{i},\text{p}}+\text{prefecture fixed effects}+\epsilon_{\text{i},\text{p}} \end{array}$$ (4)

## 4. Empirical results and discussion

### 4.1. Benchmark regression results

We measure firms’ exposure to 5A tourist attractions using the distance to the nearest 5A attraction. This choice implicitly assigns the largest weight to the closest attraction. In practice, firms may also be exposed to multiple 5A attractions, which motivates robustness checks based on alternative exposure measures. To address this, we additionally control for the median distance to all other 5A attractions (excluding the nearest) in our baseline regressions (**Dis2014_med, Dis2023_med**). To mitigate the influence of outliers, we winsorize the main continuous variables at the 1st and 99th percentiles (p1-p99), while leaving dummy variables unchanged.

Table 3 presents our baseline regression results. The results presented in Columns (1) indicate that firms closer to a 5A tourist attraction exhibit stronger environmental governance. After controlling for **Dis2014_med**, column (2) shows that the coefficient of **Dis2014** remain negative and statistically significant, reducing concerns that the estimate is driven by the spatial distribution of other attractions. Column (3) shows that, even using Conley-HAC standard errors with a 100-km cutoff, the coefficient of **Dis2014** remains significantly negative (The 100-km bandwidth spans within- and near-county interactions, thereby mitigating the risk of overstated significance due to spatially correlated shocks). Column (4) shows that, under a stricter specification with both county (company’s county-level location) and industry (finer-grained segmentation of industrial sectors) fixed effects, the negative coefficient of **Dis2014** remains robust and is slightly larger in magnitude. The results displayed in Columns (5)–(6) show that the results are robust to alternative specifications that replace the **Dis2014** with **Dis2023**. Overall, the evidence supports **Hypothesis 1**.

**Table 3:** Baseline association between proximity to 5A attractions and CEG.

	(1)	(2)	(3)	(4)	(5)	(6)
	Panel A: Main specifications				Panel B: Alternative distance measure (Dis2023)	
Dependent var	CEG	CEG	CEG	CEG	CEG	CEG
Dis2014	−0.022^***^	−0.017^*^	−0.016^*^	−0.046^**^		
	(−3.13)	(−1.74)	(−1.85)	(−2.11)		
Dis2023					−0.025^***^	−0.020^*^
					(−3.13)	(−1.84)
Size	0.001	0.001	0.001	0.002	0.002	0.002
	(0.17)	(0.16)	(0.14)	(0.20)	(0.34)	(0.35)
Subsidy	−0.109^***^	−0.109^***^	−0.109^***^	−0.109^***^	−0.107^***^	−0.107^***^
	(−11.22)	(−11.20)	(−11.14)	(−11.24)	(−11.16)	(−11.14)
Debt	0.028^***^	0.028^***^	0.028^***^	0.025^***^	0.025^***^	0.025^***^
	(6.91)	(6.91)	(7.72)	(4.48)	(5.92)	(5.90)
Nature	0.003	0.003	0.003	−0.009	0.000	−0.000
	(0.21)	(0.21)	(0.22)	(−0.46)	(0.01)	(−0.00)
lnAge	0.007	0.007	0.007	0.007	0.001	0.001
	(0.94)	(0.93)	(0.89)	(0.64)	(0.02)	(0.02)
Coast	−0.016	−0.018	−0.019	0.071	−0.009	−0.013
	(−0.87)	(−1.01)	(−1.27)	(0.08)	(−0.75)	(−0.88)
Pline	0.049^**^	0.049^**^	0.049^***^	0.091	0.049^**^	0.049^**^
	(2.58)	(2.57)	(2.96)	(0.08)	(2.54)	(2.50)
Lighting	0.011^***^	0.033	−0.191	−0.000	0.001	0.027
	(4.31)	(1.07)	(−0.72)	(−0.00)	(0.21)	(0.68)
Dis2014_med		0.210	−0.001	−0.281		
		(0.72)	(−0.01)	(−0.32)		
Dis2023_med						0.249
						(0.66)
Cons	0.897^***^	−2.505	0.574^*^	−3.710	1.197^***^	−2.805
	(7.38)	(−0.53)	(1.79)	(−0.65)	(5.47)	(−0.46)
Spatial effect (100KM)	No	No	Yes	No	No	No
Prefecture effect	Yes	Yes	Yes	No	No	No
County effect	No	No	No	Yes	Yes	Yes
Industry effect	No	No	No	Yes	Yes	Yes
SE	Clustered^a^	Clustered^a^	Clustered^a^	Clustered^a^	Clustered^a^	Clustered^a^
N	9097	9097	9097	9097	9097	9097
R^2^	0.125	0.125	0.124	0.308	0.196	0.309

Note: ***, **, and * denote statistical significance at the 1%, 5%, and 10% levels, respectively. Values in parentheses are t-statistics. Columns with “Clustered^a^” report standard errors clustered at the prefecture level.

**Fig 2:**
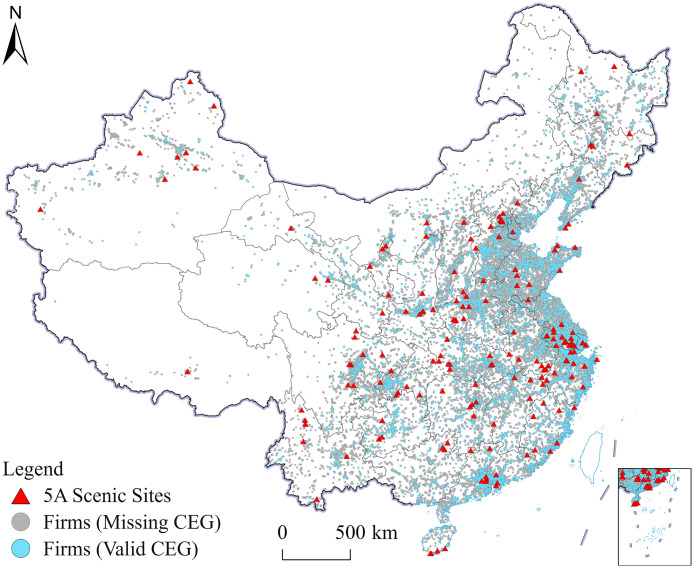
Geographic distribution of 5A tourist attraction and the enterprise sample, 2014 (The map was created using ArcGIS software. The administrative boundary data are from the National Catalogue of Geographic Information of China (http://www.webmap.cn/), which is in the public domain).

To further assess the robustness of the results of baseline, we construct an alternative measure based on the 2010 geographic distribution of 5A attractions (**Dis2010**). Columns (1)–(2) of Table 4 report negative and statistically significant coefficients on **Dis2010**, suggesting that the negative association between distance to 5A attractions and firms’ environmental governance is not driven by the attraction layout in any single year. Second, we construct **Asset_S** (enterprise accumulated depreciation divided by total capital) as a placebo variable with no direct link to tourism demand. Columns (3)–(4) indicate that the coefficients of **Dis2014** are small and statistically insignificant, alleviating concerns about spurious spatial-gradient correlation. Third, given substantial missingness in the 2014 **CEG** for industrial enterprises (18,411 non-missing observations; 93% of the sample missing), we first visualize that observed firms are geographically dispersed with no systematic clustering (Fig 2), and then impute missing CEG values as zero to obtain a conservative lower bound and estimate a logit selection model to assess robustness. Columns (5)–(6) yield estimates for **Dis2014** that are similar to the baseline results, suggesting that the association between **Dis2014** and **CEG** is not particularly sensitive to the sample restriction. Table 3 reports baseline coefficients on **Dis2014** of approximately −0.020 to −0.046. Consistent with these estimates, a 10-km increase in distance is associated with a 0.20–0.46 unit decrease in **CEG**. After introducing finer fixed effects (county and industry), the estimated magnitude increases slightly, and the inference remains in line with the spatially robust results based on Conley standard errors. Overall, results using an alternative distance measure (**Dis2010**), a placebo variable (**Asset_S**), and the two-step checks yield a similar pattern, suggesting that the main findings are qualitatively unchanged across alternative measures and sample-processing choices.

**Table 4:** Robustness checks for the baseline results.

	(1)	(2)	(3)	(4)	(5)	(6)
	Panel A: Alternative timing		Panel B: Placebo outcome (Asset_S)		Panel C: Sample restriction check (CEG with stricter filters)	
Dependent var	CEG	CEG	Asset_S	Asset_S	CEG	CEG
Dis2010	−0.041^*^	−0.022^***^				
	(−1.79)	(−3.13)				
Dis2014			2.229	2.229	−0.115^***^	−0.118^***^
			(0.30)	(0.30)	(−4.66)	(−4.26)
Controls	Yes	Yes	Yes	Yes	Yes	Yes
Dis2010_med	0.700^**^	0.212^**^				
	(2.46)	(2.22)				
Dis2014_med			−5.359	−5.359		−0.125
			(−0.72)	(−0.72)		(−0.23)
Cons	−3.844	−0.433	9.657	9.657	−1.378^**^	0.646
	(−1.01)	(−0.03)	(0.02)	(0.03)	(−3.98)	(0.07)
Prefecture effect	Yes	No	Yes	No	No	No
County effect	No	Yes	No	Yes	Yes	Yes
Industry effect	No	Yes	No	Yes	Yes	Yes
SE	Clustered^a^	Clustered^a^	Clustered^a^	Clustered^a^	Clustered^a^	Clustered^a^
N	9097	9097	139001	139001	149682	149682
R^2^	0.124	0.309	0.008	0.008	0.078	0.078

Note: ***, **, and * denote statistical significance at the 1%, 5%, and 10% levels, respectively. Values in parentheses are t-statistics. Columns with “Clustered^a^” report standard errors clustered at the prefecture level.

#### 4.1.1. Robustness testing.

Owing to data availability constraints, we were unable to obtain detailed tourism industry data for the areas surrounding each sampled industrial enterprise. Therefore, we selected the shortest geographical distance from industrial enterprises to the nearest 5A-level tourist attraction as the core explanatory variable. In practice, tourism activities are primarily concentrated in the tertiary sector, which creates a potential mismatch between the independent and dependent variables used in this study. To address this issue, we construct **TR** as the share of accommodation and food service workers in a city’s tertiary sector, measured by employment in accommodation and food services divided by total urban employment. We then use **TR** as an alternative measure of local tourism activity and re-estimate the baseline regression of **CEG** on this proxy as a robustness check.

We measure a firm’s environmental governance as wastewater treatment volume scaled by total fixed assets, reflecting treatment activity conditional on capital equipment. Nonetheless, data limitations imply that this measure may imperfectly reflect firms’ overall environmental governance. To address this concern, we consider two alternative measures as robustness checks. First, using the 2014 China Enterprise Patent Database, we match each sample firm to its total number of granted green patents and construct **Patent**, defined as the natural logarithm of this total. This measure captures a firm’s environmental governance from a technological-innovation perspective. Second, we construct **CEG_S** by dividing wastewater treatment volume by total industrial output, providing an output-adjusted measure of wastewater treatment intensity.

Columns (1)–(2) of Table 5 show that replacing the core independent variable with **TR** yields a positive and statistically significant association between accommodation and food service employment and firms’ environmental governance (**CEG**). These findings are consistent with the benchmark regression, suggesting that a larger accommodation and food services employment share (a proxy for local tourism activity) is positively associated with corporate environmental governance. Columns (3)–(6) further show that the coefficient on distance to the nearest 5A tourist attraction remains negative and statistically significant for both alternative outcomes: green patenting and wastewater treatment per unit of industrial output. The results are broadly consistent across specifications.

**Table 5:** Robustness checks using alternative exposure measures and alternative outcomes.

	(1)	(2)	(3)	(4)	(5)	(6)
Dependent var	CEG	CEG	Patent	Patent	CEG_S	CEG_S
	Panel A: Alternative Dis2014 measure (TR)		Panel B: Alternative outcome (Patent)		Panel C: Alternative CEG measure (CEG_S)	
TR	0.025^**^	0.025^***^				
	(2.55)	(10.04)				
Dis2014			−0.056^***^	−0.056^***^	−0.173^**^	−0.173^**^
			(−4.89)	(−3.08)	(−2.32)	(−2.61)
Controls	Yes	Yes	Yes	Yes	Yes	Yes
Cons	−12.830^***^	−12.830^***^	0.078	0.078	3.332^***^	3.332^***^
	(−3.04)	(−10.69)	(0.08)	(0.38)	(2.92)	(3.68)
Prefecture effect	Yes	Yes	Yes	Yes	Yes	Yes
SE	Robust	Clustered^a^	Robust	Clustered^a^	Robust	Clustered^a^
N	8771	8771	36197	36197	8992	8992
R^2^	0.122	0.122	0.092	0.092	0.045	0.045

Note: ***, **, and * denote statistical significance at the 1%, 5%, and 10% levels, respectively. Values in parentheses are t-statistics. Columns with “Clustered^a^” report standard errors clustered at the prefecture level; columns with “Robust” report heteroskedasticity-robust standard errors.

#### 4.1.2. Further discussion on the benchmark regression results.

**Fig 3:**
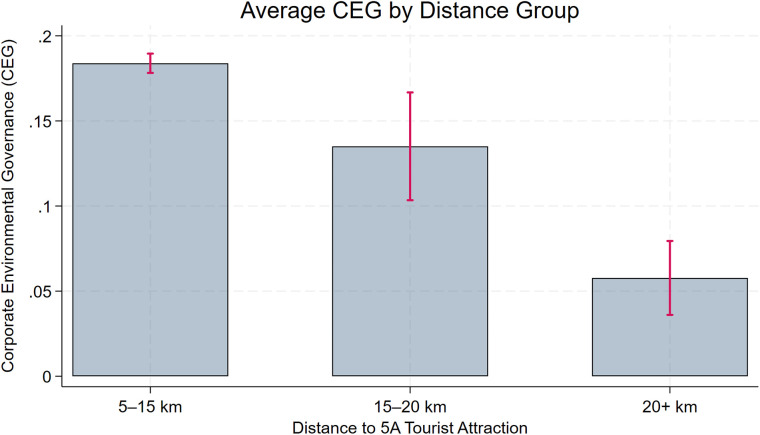
Distance-binned boxplot of CEG.

The benchmark regression yields a negative and statistically significant coefficient on **Dis2014**, implying that greater proximity to a 5A tourist attraction is associated with stronger environmental governance. However, this does not directly imply that “closer companies have stronger CEGs.” To strengthen the logical rigor of our conclusions and their empirical support, we conduct two supplementary analyses. First, we perform a distance-group regression by dividing enterprises into three groups on the basis of their proximity to the nearest 5A tourist attraction. As shown in columns (1)–(2) of Table 6, the coefficient on **Dis2014** is negative and statistically significant for the **short-distance** group, whereas the corresponding coefficients for the **medium-** and **long-distance** groups are also negative but not statistically significant. These results are consistent with the view that proximity to 5A tourist attractions is associated with stronger environmental governance. Second, we visualize group means by classifying firms into three distance bins (5–15 km, 15–20 km, and >20 km) and plotting average **CEG** with standard error bars (Fig 3). The figure shows that firms in the closest bin have higher average **CEG** than those farther away. Although the differences are modest, they are statistically significant, and the overall pattern is consistent with the benchmark regression results.

**Table 6:** Subsample regressions by distance group.

	(1)	(2)	(3)	(4)	(5)	(6)
	Short-distance group		Medium-distance group		Long-distance group	
Dependent var	CEG	CEG	CEG	CEG	CEG	CEG
Dis2014	−0.040*		−0.018		−0.022	
	(−1.91)		(−1.28)		(−0.89)	
Dis2023		−0.038*		−0.015		−0.018
		(−1.81)		(−1.16)		(−0.84)
Controls	Yes	Yes	Yes	Yes	Yes	Yes
Cons	1.135***	1.103***	1.041***	1.006***	1.039***	0.994***
	(4.13)	(4.10)	(5.20)	(5.31)	(2.99)	(3.15)
Prefecture effect	Yes	Yes	Yes	Yes	Yes	Yes
SE	Robust	Robust	Robust	Robust	Robust	Robust
N	2465	2465	3103	3103	3529	3529
R2	0.132	0.132	0.115	0.115	0.135	0.135

Note: ***, **, and * denote statistical significance at the 1%, 5%, and 10% levels, respectively. Values in parentheses are t-statistics. Columns with “Robust” report heteroskedasticity-robust standard errors.

**Fig 4:**
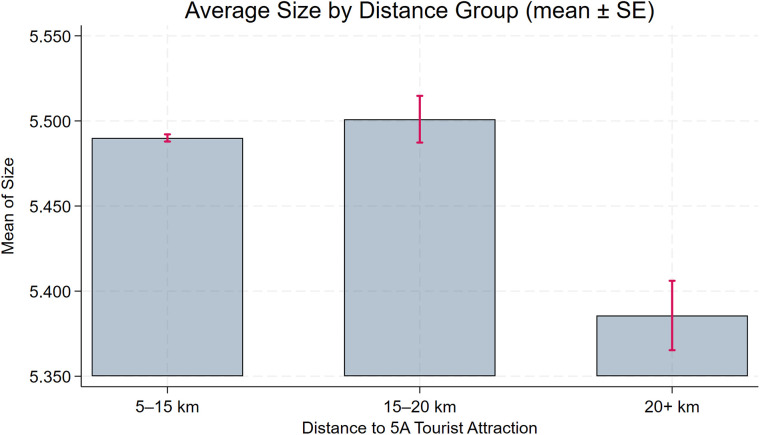
Distance-binned boxplot of Size (covariate balance).

**Fig 5:**
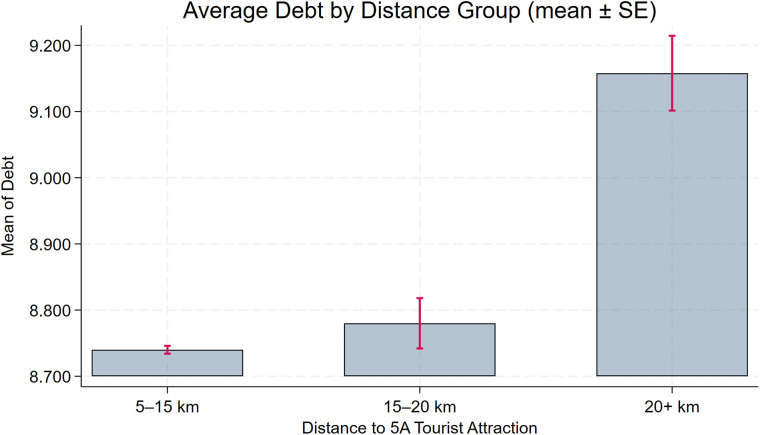
Distance-binned boxplot of Debt (covariate balance).

**Fig 6:**
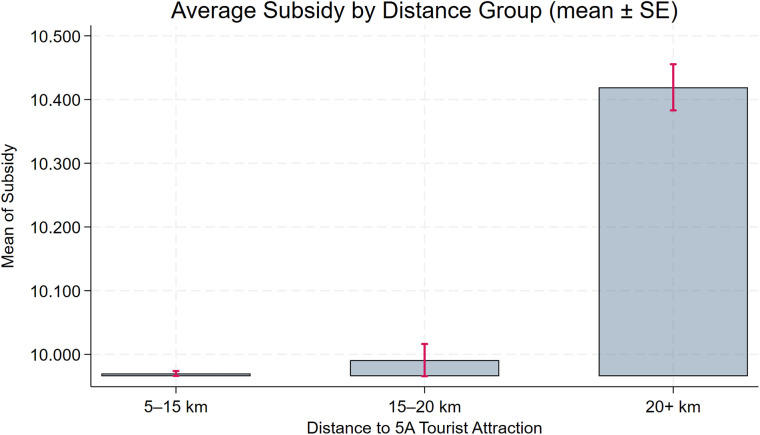
Distance-binned boxplot of Subsidy (covariate balance).

**Fig 7:**
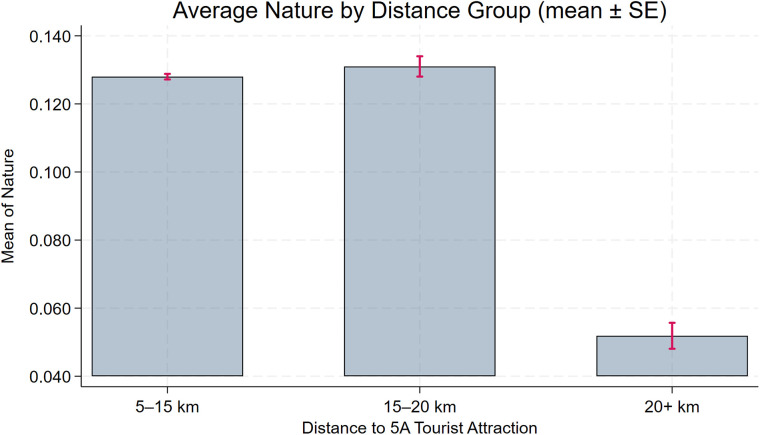
Distance-binned boxplot of Nature (covariate balance).

**Fig 8:**
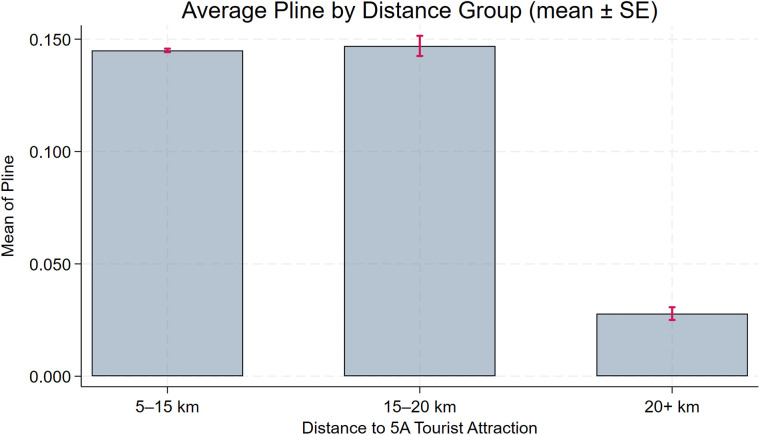
Distance-binned boxplot of Pline (covariate balance).

**Fig 9:**
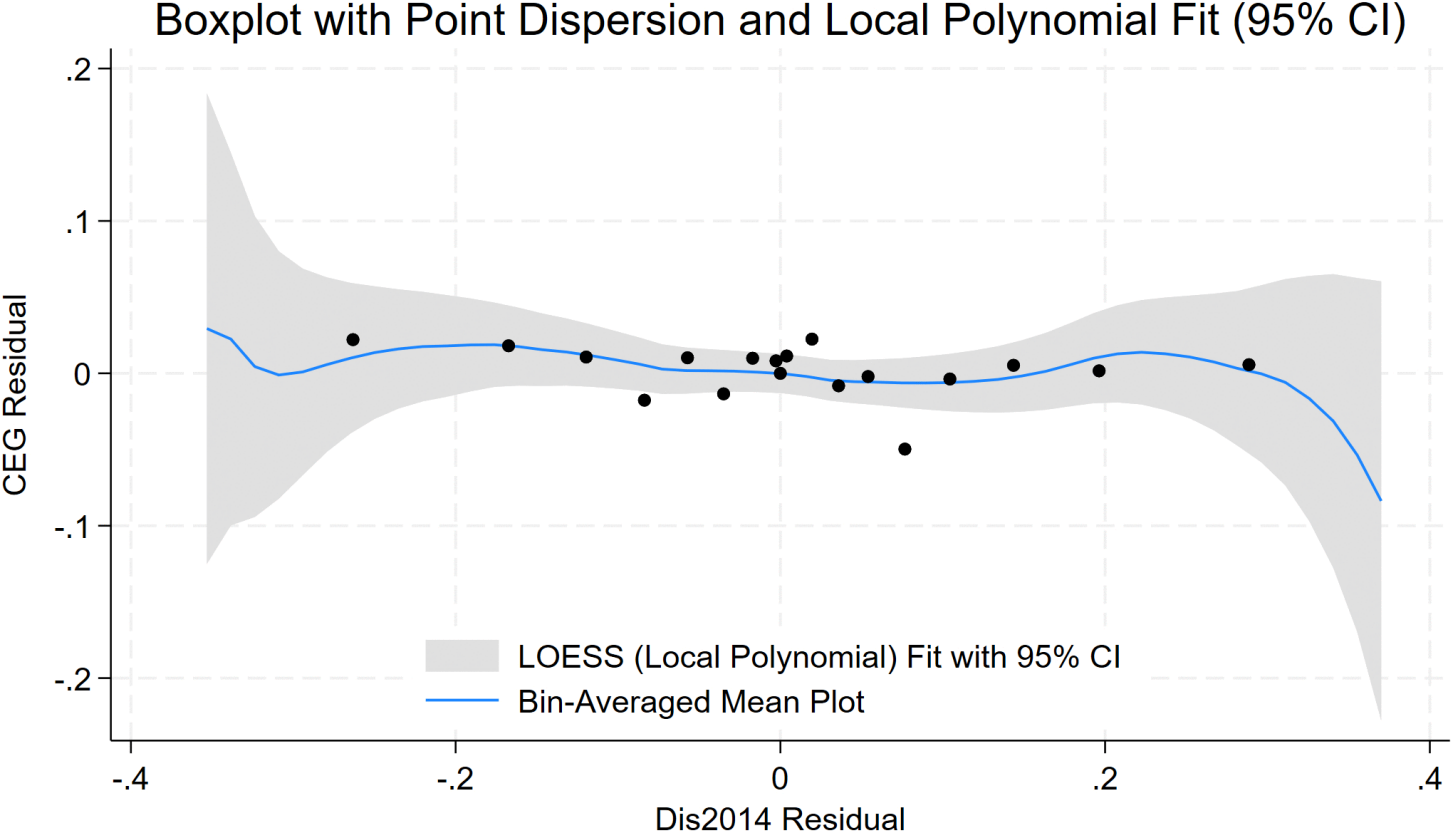
Residualized CEG versus distance: binscatter with local polynomial fit.

**Fig 10:**
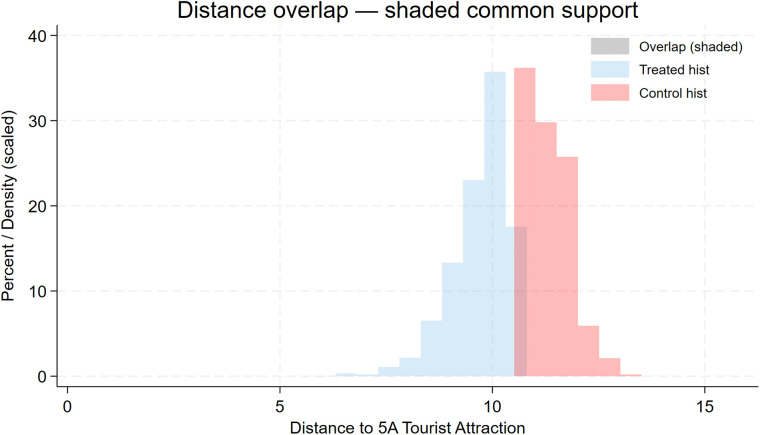
Distance distributions around the median: assessing common support (near vs. far firms).

To assess the robustness of the baseline model, we further plot distance-binned boxplots for the key control variables (**Size**, **Debt**, **Subsidy**, **Nature**, **Pline**) to evaluate covariate balance. Figs 4, 5, 6, 7, 8 show that, for each control variable, the distance-binned boxplot means vary only slightly with distance to the 5A tourist attraction. The two groups closest to the attraction are similar in levels, with only minor differences, indicating that sample-selection bias is unlikely in the baseline regression. Then, we regress **CEG** on the full set of controls and fixed effects, obtain the residuals, and plot a binscatter with a local polynomial fit using these residuals. Fig 9 shows a clear downward pattern in residualized CEG as distance increases, indicating that, after conditioning on covariates and fixed effects, **CEG** remains negatively associated with distance. Finally, we split firms into “near” and “far” groups based on whether their distance to the nearest 5A tourist attraction is below or above the sample median, and assess common support using a distance histogram. Fig 10 shows substantial overlap between the two distributions around the median, with only limited mass in the tails, suggesting reasonable common support and a similar distance range across the two groups.

#### 4.1.3. Placebo tests.

**Fig 11:**
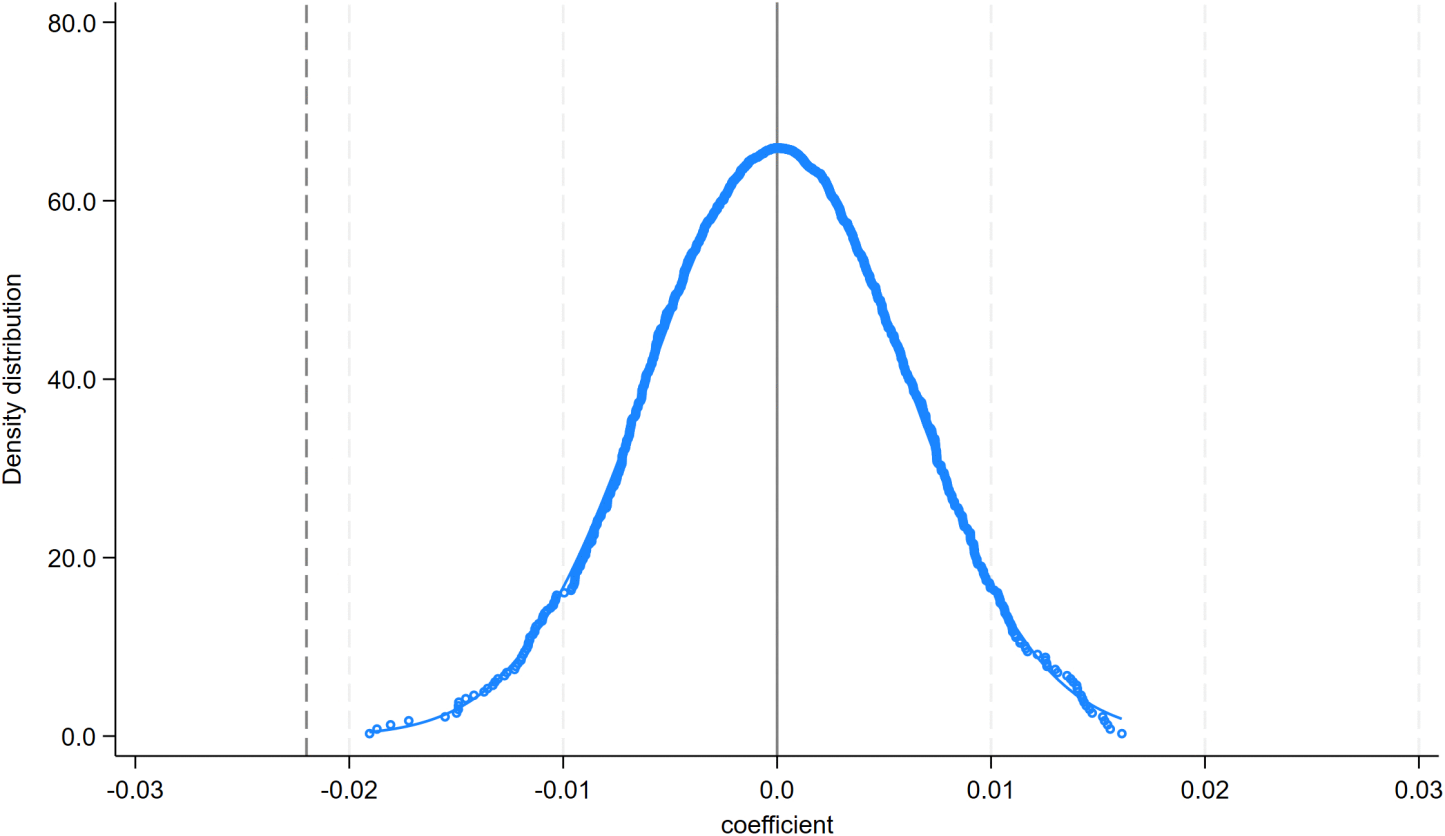
Distribution of placebo coefficients: Dis2014.

**Fig 12:**
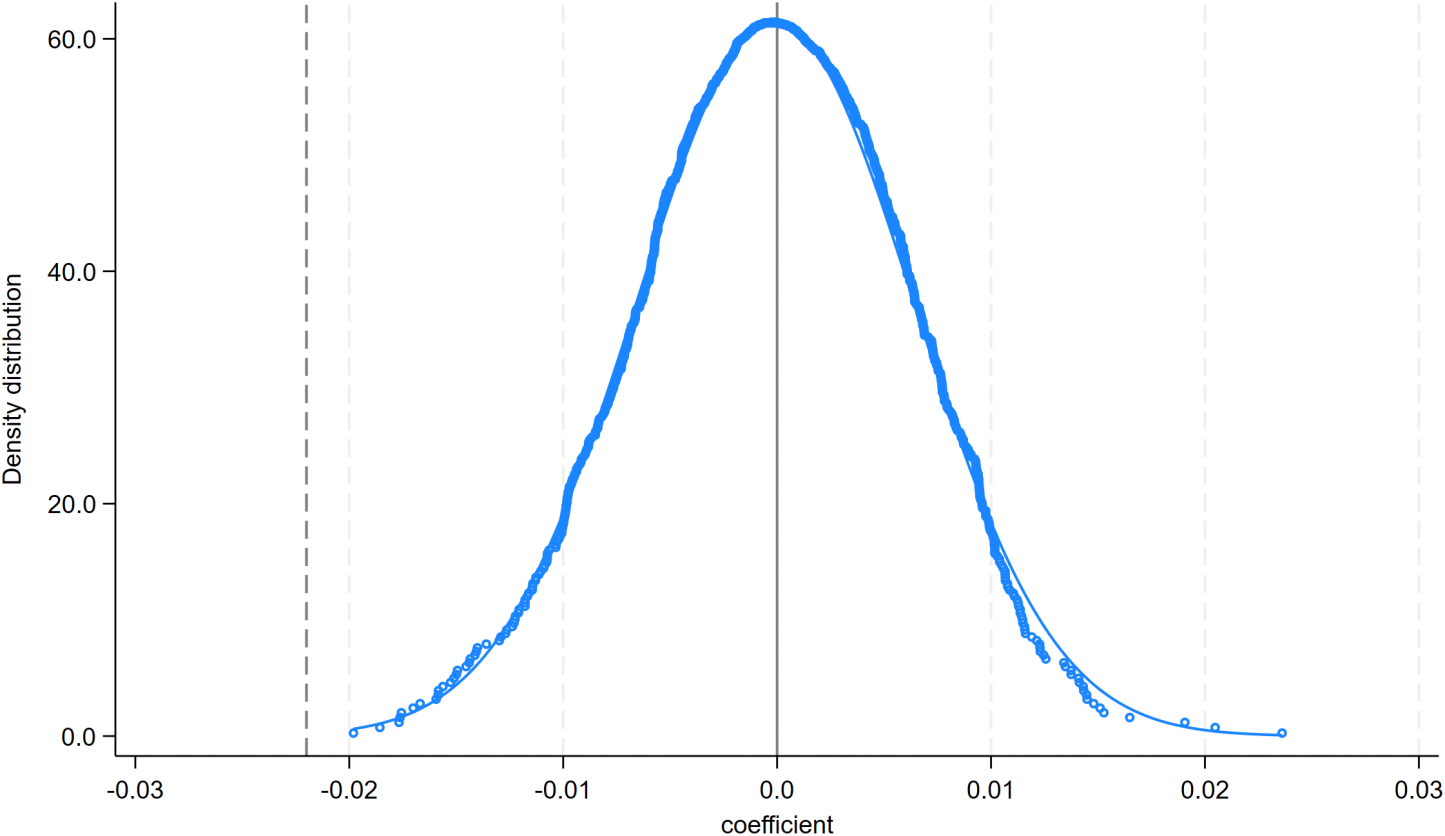
Distribution of placebo coefficients: Dis2023.

Furthermore, we conduct placebo tests by repeating the regression 1,000 times using randomly generated (placebo) values of **Dis2014** and **Dis2023**. The distribution of placebo coefficients is centered around zero and is rarely statistically significant, indicating that the baseline estimates are unlikely to be driven by chance (Figs 11 and 12). Overall, the placebo evidence is consistent with **Hypothesis 1.**

### 4.2. Instrumental variable (IV) estimation

#### 4.2.1 IV robustness checks: baseline and channel results.

During the matching process, we found that many industrial enterprises reported zero or missing wastewater discharge data. This may reflect non-reporting by the surveying agency or production processes that generate little or no wastewater. Because such missingness may be non-random, our baseline estimates could be affected by sample selection and other unobserved confounders. To further assess whether our baseline association is robust to potential endogeneity, we implement an instrumental-variables (IV) estimation as a robustness check. We use terrain **slope** at the city level as an instrument. Terrain is an important determinant of tourism resource endowments. Cities with steeper or more rugged terrain are more likely to host prominent scenic resources and sites that are subsequently designated as 5A tourist attractions. Accordingly, city-level slope should be correlated with firms’ proximity to 5A tourist attractions.

Because our analysis relies on cross-sectional data, the IV strategy cannot deliver definitive causal identification. We therefore use city-level terrain slope in a 2SLS framework as a robustness check for potential endogeneity. The exclusion restriction may be violated if slope directly affects environmental governance through drainage conditions, wastewater dispersion, infrastructure and abatement costs, or industrial siting patterns. Accordingly, we interpret the IV estimates as suggestive rather than conclusive.

Table 7 reports the 2SLS estimates. Columns (1)–(2) show that city-level terrain **slope** is statistically associated with **Dis2014** and **Dis2023**, which is consistent with the relevance condition. Columns (3)–(4) report an informal direct-effect check by regressing **CEG** on **slope** while conditioning on the distance measures. The slope coefficients are small and statistically indistinguishable from zero, suggesting that **slope** does not exhibit a residual association with **CEG** once distance is included. We emphasize, however, that this exercise is not a formal test of the exclusion restriction, and slope may still affect firms’ environmental governance through other channels discussed above. Columns (5)–(6) present the second-stage estimates. The coefficients on the proximity measures remain qualitatively similar to the baseline association, suggesting that firms located closer to 5A attractions tend to exhibit stronger environmental governance on average. The IV results are broadly in line with our baseline findings for **Hypothesis 1**.

**Table 7:** IV estimates (CEG).

	Panel A: First stage		Panel B: Direct-effect check		Panel C: Second stage (2SLS)	
	(1)	(2)	(3)	(4)	(5)	(6)
Dependent var.	Dis2014	Dis2023	CEG	CEG	CEG	CEG
Slope	−0.002***	−0.002***	0.019	0.023		
	(−4.84)	(−4.21)	(0.24)	(0.35)		
Dis2014			−0.020**		−5.292***	
			(−2.02)		(−4.38)	
Dis2023				−0.020**		−6.949***
				(−2.53)		(−3.47)
Size	0.033***	0.037***	0.001	0.001	0.166***	0.245**
	(3.72)	(4.06)	(0.24)	(0.23)	(2.70)	(2.57)
Subsidy	−0.004	0.001	−0.112***	−0.112***	−0.116***	−0.087*
	(−0.70)	(0.14)	(−11.30)	(−11.28)	(−3.50)	(−2.00)
Debt	−0.001	−0.005	0.029***	0.029***	0.019	−0.011
	(−0.23)	(−1.05)	(6.90)	(6.88)	(0.73)	(−0.30)
Nature	−0.114***	−0.132***	0.004	0.004	−0.606***	−0.922***
	(−5.45)	(−6.18)	(0.32)	(0.30)	(−3.36)	(−2.99)
lnAge	−0.098***	−0.108***	0.005	0.005	−0.516***	−0.748***
	(−10.12)	(−11.04)	(0.69)	(0.65)	(−3.94)	(−3.26)
Coast	0.369***	0.298***	−0.009	−0.009	1.923***	2.041***
	(16.83)	(13.83)	(−0.43)	(−0.42)	(4.21)	(3.35)
Pline	0.233***	0.296***	0.045**	0.045**	1.253***	2.077***
	(12.25)	(14.54)	(2.20)	(2.22)	(4.23)	(3.44)
Lighting	−0.035***	−0.027***	−0.005	−0.005	−0.187***	−0.185***
	(−69.73)	(−52.46)	(−1.00)	(−1.48)	(−4.38)	(−3.47)
Dis2014_med	0.069		−0.022		0.415*	
	(1.55)		(−0.06)		(1.64)	
Dis2023_med		0.232***		−0.049		1.664***
		(4.59)		(−0.19)		(2.84)
Cons	10.258***	7.573***	1.496	1.875	54.462***	52.779***
	(16.70)	(10.81)	(0.31)	(0.51)	(4.28)	(3.34)
First-stage F-statistic	22.37	18.57				
Prefecture effect	Yes	Yes	Yes	Yes	Yes	Yes
SE	Roubst	Roubst	Roubst	Roubst	Roubst	Roubst
N	9097	9097	9097	9097	9097	9097

Note: ***, **, and * denote statistical significance at the 1%, 5%, and 10% levels, respectively. Values in parentheses are t-statistics. Columns with “Robust” report heteroskedasticity-robust standard errors.

We further examine a potential channel by estimating the 2SLS specification with the fixed-asset ratio (**CFI**) as the outcome. Our hypothesis is that firms located closer to 5A attractions may exhibit higher fixed-asset intensity, which is consistent with stronger environmental governance. Table 8 reports the results. Columns (5)–(6) present the second-stage 2SLS estimates. The coefficient on **Dis2014** remains statistically significant and indicates that firms closer to 5A attractions tend to have higher fixed-asset ratios on average. Taken together, these results are consistent with the channel hypothesis that proximity to 5A attractions is associated with higher fixed-asset intensity.

**Table 8:** IV estimates (CFI).

	Panel A: First stage		Panel B: Direct-effect check		Panel C: Second stage (2SLS)	
	(1)	(2)	(3)	(4)	(5)	(6)
Dependent var.	Dis2014	Dis2023	CFI	CFI	CFI	CFI
Slope	−0.003^***^	−0.002^***^	0.034	−0.011		
	(−4.41)	(−4.61)	(0.27)	(−0.09)		
Dis2014			−0.079^**^		−1.112^***^	
			(−2.58)		(−3.61)	
Dis2023				−0.074^***^		−1.298^***^
				(−2.71)		(−3.51)
Size	0.046^***^	0.042^***^	0.110^***^	0.110^***^	0.116^***^	0.119^***^
	(3.42)	(3.08)	(9.26)	(9.26)	(3.31)	(3.25)
Subsidy	−0.003	−0.002	0.494^***^	0.494^***^	0.548^***^	0.549^***^
	(−0.30)	(−0.22)	(47.67)	(47.57)	(24.18)	(23.30)
Debt	−0.005	−0.004	0.094^***^	0.094^***^	0.116^***^	0.115^***^
	(−0.62)	(−0.60)	(10.29)	(10.26)	(6.43)	(6.13)
Nature	−0.084^***^	−0.082^***^	−0.049	−0.050	−0.110	−0.122
	(−2.80)	(−2.71)	(−1.10)	(−1.11)	(−1.44)	(−1.51)
lnAge	−0.097^***^	−0.092^***^	−0.021	−0.022	−0.045	−0.057
	(−6.78)	(−6.37)	(−1.11)	(−1.14)	(−0.94)	(−1.12)
Coast	0.417^***^	0.343^***^	0.006	0.013	0.377^**^	0.363^**^
	(13.02)	(11.04)	(0.12)	(0.26)	(2.56)	(2.46)
Pline	0.220^***^	0.311^***^	0.054	0.053	0.102	0.265^*^
	(7.71)	(10.30)	(0.94)	(0.93)	(1.02)	(1.93)
Lighting	−0.035^***^	−0.026^***^	0.080^***^	0.085^***^	−0.033^***^	−0.028^***^
	(−45.85)	(−34.40)	(10.78)	(10.50)	(−3.04)	(−2.90)
Dis2014_med	−0.225^***^	−0.050	−1.187^*^		−1.063^***^	
	(−2.85)	(−0.53)	(−1.97)		(−5.87)	
Dis2023_med				−0.888		−0.951^***^
				(−1.33)		(−4.88)
Cons	14.238^***^	11.373^***^	18.196^**^	13.920	28.362^***^	28.367^***^
	(13.15)	(8.67)	(2.10)	(1.46)	(5.75)	(5.69)
First-stage F-statistic	23.68	16.99				
Prefecture effect	Yes	Yes	Yes	Yes	Yes	Yes
SE	Roubst	Roubst	Roubst	Roubst	Roubst	Roubst
N	9097	9097	9097	9097	9097	9097

Note: ***, **, and * denote statistical significance at the 1%, 5%, and 10% levels, respectively. Values in parentheses are t-statistics. Columns with “Robust” report heteroskedasticity-robust standard errors.

#### 4.2.2 Sensitivity checks and OLS–IV comparisons.

Table 9 reports additional sensitivity checks for the 2SLS specification. Columns (1)–(4) show that city-level slope remains a strong predictor of **Dis2014** in the first stage. The **slope** coefficient is statistically significant, and the first-stage F-statistics range from 21 to 23, mitigating weak-instrument concerns. The estimated relationship is stable when we add a set of geographic controls (**Inside** and **Touch** are constructed from administrative-boundary adjacency, where **Inside** captures proximity to provincial borders, the coastline, or national borders, and **Touch** captures proximity to land borders. **North** is defined using a standard north-south division of China (e.g., the Qinling–Huaihe line). Industry fixed effects are defined at the **[2-digit]** industry level based on firms’ primary industry codes).Specifically, **Inside** is an indicator for counties adjacent to a provincial boundary, the coastline, or a national border; **Touch** indicates whether a county is adjacent to a land border; and **North** indicates whether a county is located in northern China. Column (4) further includes industry fixed effects. Taken together, these results suggest that the first-stage association is not mechanically driven by border attributes, north-south differences, or industry composition. Column (5) reports the second-stage estimate with these additional controls, and the coefficient on Dis2014 remains in the same direction as in the baseline models.

**Table 9:** Sensitivity checks for the IV estimates.

	Panel A: First stage				Panel B: Second stage (2SLS)
	(1)	(2)	(3)	(4)	(5)
Dependent var.	Dis2014	Dis2014	Dis2014	Dis2014	CEG
Slope	−0.002^***^	−0.002^***^	−0.002^***^	−0.002^***^	
	(−4.87)	(−4.82)	(−4.80)	(−4.83)	
Dis2014					−5.217^***^
					(−4.53)
Inside	−0.220^***^	0.196^***^	0.188^***^	0.203^***^	
	(−4.11)	(4.58)	(4.43)	(4.28)	
Touch		1.261^***^	1.247^***^	1.136^***^	
		(16.95)	(16.65)	(13.64)	
North			−0.087^***^	−0.082^***^	
			(−4.08)	(−3.61)	
Industry effect	No	No	No	Yes	Yes
Controls	Yes	Yes	Yes	Yes	Yes
Cons	10.781^***^	12.111^***^	12.516^***^	13.618^***^	71.468^***^
	(17.18)	(18.42)	(19.46)	(19.80)	(4.47)
First-stage F-statistic	22.546	22.056	21.230	22.978	
Prefecture effect	Yes	Yes	Yes	Yes	Yes
SE	Roubst	Roubst	Roubst	Roubst	Roubst
N	9097	9097	9097	9097	9097

Note: ***, **, and * denote statistical significance at the 1%, 5%, and 10% levels, respectively. Values in parentheses are t-statistics. Columns with “Robust” report heteroskedasticity-robust standard errors.

These checks do not validate the exclusion restriction, but they help assess whether the IV results are sensitive to observable geographic and industry-related channels through which **slope** could directly affect environmental governance. With these controls, the IV estimates remain broadly consistent with the baseline association, and we therefore interpret them as suggestive rather than definitive.

Table 10 compares OLS and 2SLS estimates using both the raw CEG measure and its standardized counterpart. Using the raw outcome, the 2SLS coefficient on **Dis2014** is −5.292 (Table 7 and Table 10, Column (2)), whereas the corresponding OLS coefficient is −0.022 (Table 10, Column (1)). The first-stage F-statistic is 22.37, mitigating weak-instrument concerns. We interpret the 2SLS magnitude cautiously given the cross-sectional design and the possibility of alternative channels linking **slope** to **CEG**, as discussed above.

**Table 10:** OLS and 2SLS estimates using raw and standardized CEG.

	(1)	(2)	(3)	(4)
	Panel A: Raw CEG		Panel B: Standardized CEG	
	OLS	2SLS (IV = Slope)	OLS	2SLS (IV = Slope)
Dependent var	CEG	CEG	CEG_std	CEG_std
Dis2014	−0.022**	−5.292***	−0.030**	−7.062***
	(0.011)	(1.207)	(0.015)	(1.611)
Controls	Yes	Yes	Yes	Yes
Prefecture effect	Yes	Yes	Yes	Yes
SE	Robust	Robust	Robust	Robust
N	9097	9097	9097	9097
R2	0.124	—	0.124	—
First-stage F-statistic		22.37		23.39

Note: ***, **, and * denote statistical significance at the 1%, 5%, and 10% levels, respectively. Values in parentheses are t-statistics. Columns with “Robust” report heteroskedasticity-robust standard errors.

Because **CEG** is defined as wastewater treatment volume scaled by total fixed assets, its level can be sensitive to rescaling (mean = 0.181; SD = 0.749). Consistent with this, re-estimating the model using standardized CEG yields a statistically significant effect with the same sign (**CEG_std**: −7.062; Table 10, Column (4)). Finally, even under standard IV assumptions, 2SLS identifies a local relationship for compliers; with heterogeneous effects, its magnitude need not coincide with the OLS estimate [62].

## 5. Mechanism testing

### 5.1. Mediation analysis via operating costs and fixed-asset intensity

To explore potential channels through which proximity to 5A attractions is associated with firms’ environmental governance, we implement a causal mediation analysis following the framework in [63,64]. Using Equations (2)–(3), we decompose the total association into an indirect component operating through the mediator (**Cost** or **CFI**) and a direct component, and report the average causal mediation effect (ACME). In practice, we estimate the mediator and outcome models specified in Equations (2)–(3) and compute ACME based on simulated counterfactual predictions [65]. Given the cross-sectional design, these mediation results should be interpreted as suggestive evidence conditional on the identifying assumptions of the mediation framework, rather than definitive causal mechanisms.

### 5.2. Addressing measurement and confounding concerns

Due to data limitations, our operating-cost measure (**Cost**) aggregates multiple expense categories and may not cleanly capture transportation-related cost changes. We therefore complement the mechanism discussion with two proxies more directly linked to traffic accessibility: **lnSco**, defined as the logarithm of firms’ main-business product sales, and **lnHwy**, defined as the logarithm of the distance from each firm to its nearest primary highway toll station. We obtain the coordinates of primary highway toll stations from China’s 2014 National Toll Road Statistical Bulletin and compute the great-circle distance between each firm and its nearest primary toll station using R. The resulting distance is log-transformed to construct **lnHwy**. The Bulletin reports 1,665 mainline toll stations nationwide by end-2014, including 442 on primary highways.

These auxiliary regressions help assess whether the baseline patterns are consistent with a traffic-accessibility interpretation. We also note that lower operating costs do not necessarily capture firms’ governance incentives; instead, they may be related to governance capacity through capital expenditures. Accordingly, we examine the fixed asset-to-asset ratio (**CFI**) as an additional channel outcome. To reduce confounding from production expansion, we control for firms’ operating scale and investment intensity by including **lnRoi** (returns on investment), **lnInt** (inventories), and **lnSrn** (main-business revenue) in the regressions.

Columns (1)–(2) of Table 11 report auxiliary regressions using two proxies for traffic accessibility. **Dis2014** is positively associated with **lnHwy** and **lnSco**, indicating that firms located closer to 5A attractions tend to be closer to primary highway toll stations and exhibit lower transport-related frictions as captured by these measures. Taken together, these patterns are consistent with a traffic-accessibility interpretation in which areas around 5A attractions are better connected.

**Table 11:** Evidence on channels: infrastructure and compliance-related investment.

	(1)	(2)	(3)	(4)	(5)
	Panel A: Traffic accessibility		Panel B: Compliance-related investment		
Dependent var	lnHwy	lnSco	CFI	CFI	CFI
Dis2014	0.029***	0.014***	−0.086*	−0.100**	−0.091**
	(10.40)	(2.67)	(−1.95)	(−2.33)	(−2.09)
Controls	Yes	Yes	Yes	Yes	Yes
lnRoi			0.136***	0.113***	0.108***
			(12.40)	(10.40)	(9.55)
lnInt				0.217***	0.226***
				(12.98)	(12.67)
lnSrn					−0.039
					(−1.64)
Cons	14.777^***^	2.458^***^	3.922^**^	3.379^*^	3.397^*^
	(444.47)	(38.31)	(2.42)	(1.82)	(1.75)
Prefecture effect	Yes	Yes	Yes	Yes	Yes
SE	Robust	Robust	Robust	Robust	Robust
N	150105	150076	4992	4981	4830
R^2^	0.911	0.496	0.456	0.489	0.479

Note: ***, **, and * denote statistical significance at the 1%, 5%, and 10% levels, respectively. Values in parentheses are t-statistics. Columns with “Robust” report heteroskedasticity-robust standard errors.

Columns (3)–(5) examine fixed-asset intensity (**CFI**). The coefficient on **Dis2014** remains statistically significant after controlling for proxies of production expansion (**lnRoi**, **lnInt**, and **lnSrn**). Given that **Dis2014** is a distance measure, the negative coefficient implies that firms closer to 5A attractions tend to have higher fixed-asset intensity on average, and this relationship is not mechanically driven by differences in expansion-related firm characteristics.

In our framework, fixed-asset intensity is not meant to capture governance intentions directly. Instead, it proxies for firms’ capacity to undertake environmental governance through capital expenditures. Under stronger institutional pressure and reputational concerns, firms may upgrade or expand physical assets that facilitate compliance and abatement. Therefore, while fixed assets are a broad category, the observed association provides suggestive support for the capacity-based channel in areas near 5A attractions.

### 5.3. Results of mediation analysis

Panel A of Table 12 reports exploratory mediation and decomposition estimates. For **Dis2014**, the total effect on **CFI** is negative and statistically significant (−0.129, p < 0.01), indicating that firms located farther from 5A attractions tend to exhibit lower fixed-asset intensity on average. A similar pattern holds for Dis2023 (total effect = −0.124, p < 0.01).

**Table 12:** Exploratory mediation and decomposition results.

Panel A. Decomposition (I): Mediator = Cost; Outcome = CFI (without controlling for lnCap)		
	Dis2014	Dis2023
Average causal mediation effect (ACME)	0.012***	0.021***
Direct effect	−0.142***	−0.145***
Total effect	−0.129***	−0.124***
Share of total effect mediated	−0.090***	−0.166***
**Panel B.** Decomposition (II): Mediator = **Cost**; Outcome = **CFI** (controlling for lnCap)		
	Dis2014	Dis2023
Average causal mediation effect (ACME)	−0.012**	−0.002*
Direct effect	−0.011***	−0.008**
Total effect	−0.023***	−0.011*
Share of total effect mediated	0.524***	0.189*
**Panel C.** Decomposition (III): Mediator = **CFI**; Outcome = **CEG**		
	Dis2014	Dis2023
Average causal mediation effect (ACME)	−0.003***	−0.003***
Direct effect	−0.020*	−0.014
Total effect	−0.025**	−0.016
Share of total effect mediated	0.115***	0.157

Note: This table reports exploratory cross-sectional mediation and decomposition results. ACME denotes the average causal mediation effect. “Share of total effect mediated” equals ACME divided by the total effect (and can exceed 1 or be negative when ACME and total effects differ in sign). ***, **, and * denote statistical significance at the 1%, 5%, and 10% levels, respectively.

The decomposition further shows a positive indirect component operating through **Cost** (ACME = 0.012 for **Dis2014** and 0.021 for **Dis2023**, both p < 0.01), while the direct component remains negative (−0.142 and −0.145, respectively). This sign reversal implies an “inconsistent” (competitive) mediation pattern: the cost-related channel offsets part of the negative total association rather than reinforcing it. Consistent with this, the share mediated is negative (−9.0% for **Dis2014** and −16.6% for **Dis2023**), suggesting that the **Cost** pathway partially counteracts the overall negative association between distance and fixed-asset intensity.

The sign reversal in the benchmark mediation results may reflect that **Cost** partly captures firm scale or capital intensity. Larger firms tend to incur higher operating costs but may also have stronger investment capacity, which can mechanically generate a positive indirect component. To reduce this scale-related confounding, we add paid-in capital as a control in the mediation models. Paid-in capital is measured as the logarithm of a firm’s annual paid-in capital (**lnCap**).

Panel B of Table 12 shows that, after controlling for **lnCap**, the estimated indirect component through **Cost** (ACME) becomes negative (−0.012, p < 0.05 for **Dis2014**; −0.002, p < 0.10 for **Dis2023**). This pattern is consistent with a cost-based channel in which greater distance is associated with higher operating costs and, in turn, lower fixed-asset intensity. The implied mediated share is sizeable (52.4% for **Dis2014** and 18.9% for **Dis2023**). Interpreted with caution, the mediation results suggest that the **Cost** pathway may account for a non-trivial portion of the overall association highlighted in **Hypothesis 2**.

To explore whether fixed-asset intensity (**CFI**) is a potential channel linking proximity to 5A attractions and environmental governance (**CEG**), Panel C of Table 12 reports mediation estimates with **CFI** as the mediator and **CEG** as the outcome. For **Dis2014**, the estimated indirect component through **CFI** is negative and statistically significant (ACME = −0.003, p < 0.01), consistent with the idea that firms farther from 5A attractions tend to have lower fixed-asset intensity, which is in turn associated with weaker wastewater treatment capacity. The direct component is also negative (−0.020, p < 0.10), and the total effect is negative and statistically significant (−0.025, p < 0.05). The implied mediated share is about 11.5%. Overall, the pattern is consistent with **CFI** being one observable correlate of the relationship between proximity to 5A attractions and **CEG**, and it provides supportive but not definitive evidence for **Hypothesis 3**.

### 5.4. Heterogeneity analysis

#### 5.4.1. Heterogeneity by attraction type.

We examine whether the relationship between proximity to 5A attractions and firms’ environmental governance varies with the type of attraction. We classify 5A attractions as natural or cultural and define a dummy variable, **Lands**, which equals one for natural attractions and zero otherwise, based on the descriptions provided on the official website of the Ministry of Culture and Tourism of China.

**Fig 13:**
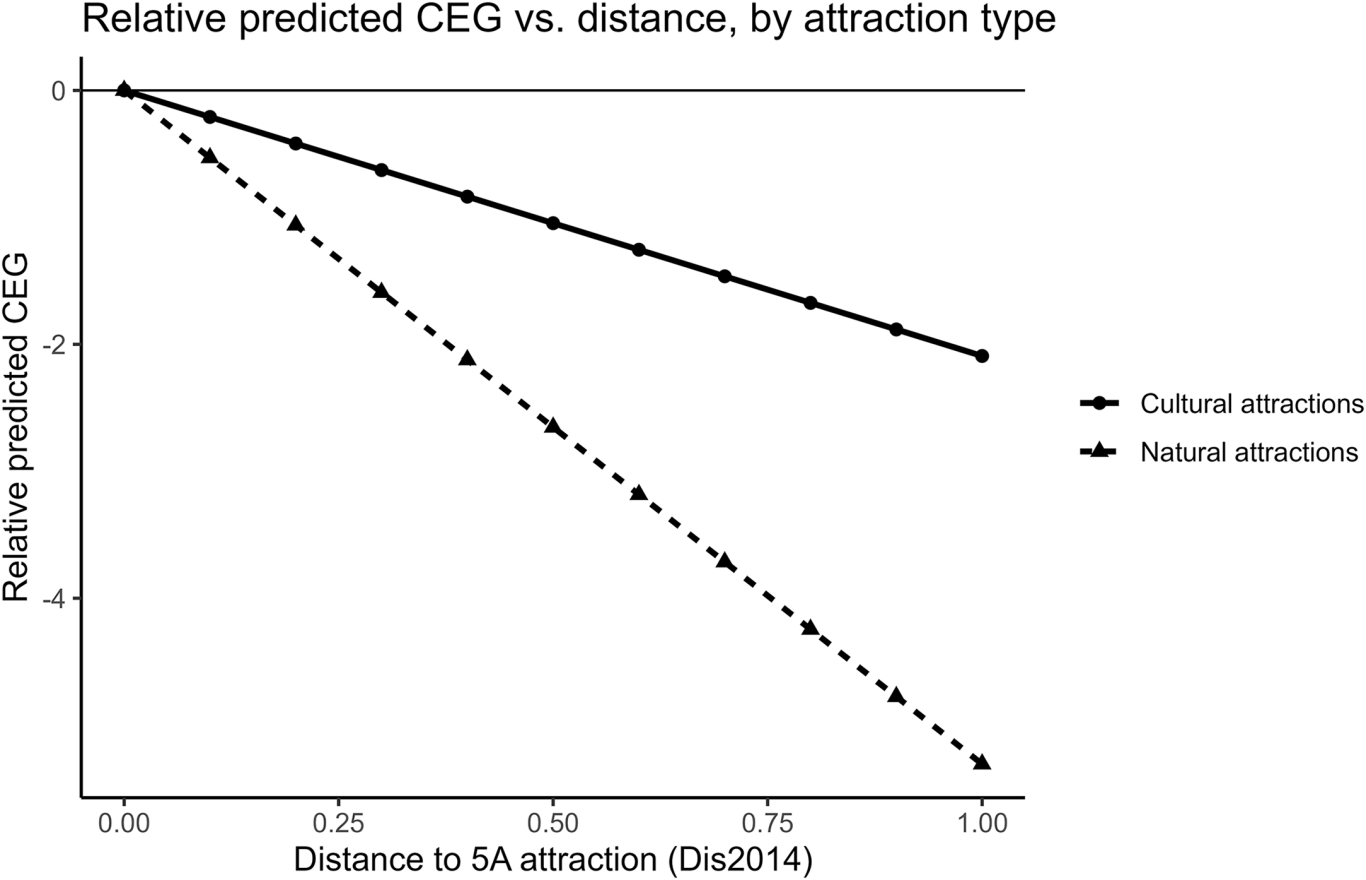
Relative predicted CEG versus distance, by attraction type.

We estimate Equation (4) by interacting the distance measure with **Lands**. Consistent with our baseline IV strategy, we implement a 2SLS specification in which we instrument both **Dis2014** and the interaction term **Dis2014 × Lands** using the terrain-slope instrument and its interaction with Lands (**Slop** and **Slop×Lands**). Columns (1)–(2) of Table 13 report the first-stage results, which indicate that the instruments are correlated with the corresponding endogenous regressors. Column (3) reports the second-stage estimates. To make the heterogeneity more transparent, Fig 13 plots the implied relative predicted CEG-distance profiles separately for natural and cultural attractions (normalized to zero at distance = 0 within each group). The coefficient on **Dis2014 × Lands** is statistically significant, suggesting that the association between distance to 5A attractions and **CEG** differs between natural and cultural attractions. In particular, the estimates imply that the relationship between proximity to 5A attractions and CEG is stronger in counties with natural 5A attractions than in counties with cultural attractions.

**Table 13:** Attraction type and environmental governance.

	(1)	(2)	(3)
	Panel A: First stage		Panel B: Second stage (2SLS)
Dependent var	Dis2014	Dis2014 × Lands	CEG
Dis2014			−2.092***(−2.92)
Dis2014 × Lands			−3.214***(−3.37)
Slope	−0.044(−1.54)	0.024*(1.67)	
Slope×Lands	0.041*(1.87)	−0.027*(−1.83)	
Lands	0.089(1.51)	10.510***(248.73)	33.970***(3.40)
Controls	Yes	Yes	Yes
Cons	10.154***(16.42)	−0.107(−0.17)	21.072***(2.72)
Prefecture effect	Yes	Yes	Yes
SE	Robust	Robust	Robust
N	9097	9097	9097

Note: ***, **, and * denote statistical significance at the 1%, 5%, and 10% levels, respectively. Values in parentheses are t-statistics. Columns with “Robust” report heteroskedasticity-robust standard errors.

Given the cross-sectional design and the potential threats to the exclusion restriction, we interpret this heterogeneity pattern as suggestive. One possible interpretation is that natural attractions are more likely to be accompanied by environmental-amenity investments and regulatory attention, which may be associated with greater environmental compliance efforts and governance capacity among nearby firms. This evidence is broadly consistent with **Hypothesis 4**. We next examine auxiliary Hypotheses 4.1 and 4.2.

#### 5.4.2. Heterogeneity by urban innovation.

We examine heterogeneity by urban innovation intensity. We define **Innovation** as an indicator equal to one if a city’s innovation expenditure to GDP ratio in 2014 (innovation expenditure divided by GDP) is at or above the sample median across all cities, and zero otherwise. We then estimate Equation (4) by interacting **Dis2014** with **Innovation**.

**Fig 14:**
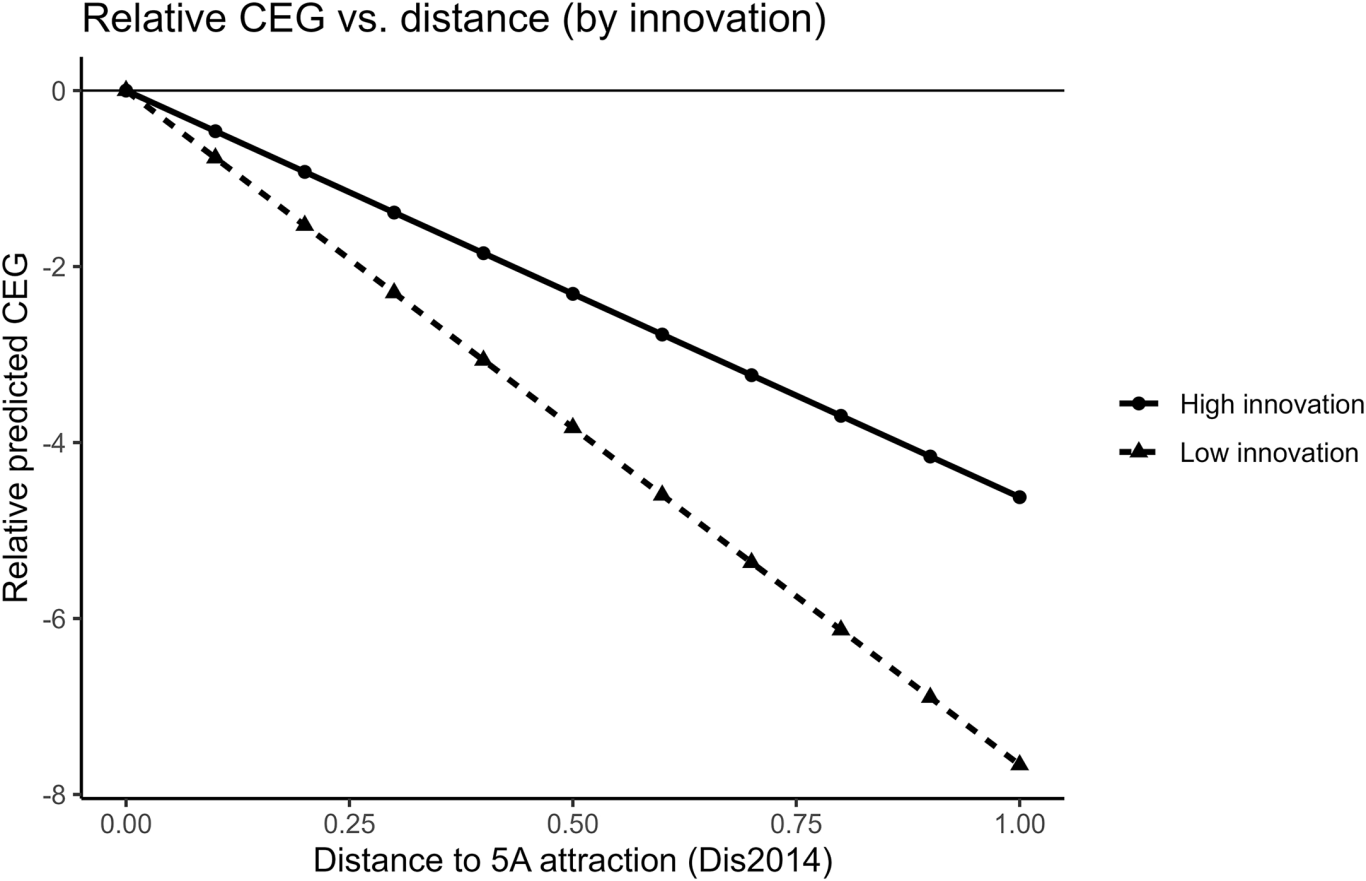
Relative predicted CEG versus distance, by urban innovation intensity.

Following the approach in Table 13, we implement a 2SLS specification in which both **Dis2014** and **Dis2014** × **Innovation** are instrumented using the terrain-slope instrument and its interaction with **Innovation** (**Slop** and **Slop**×**Innovation**). Table 14 reports the results. Column (3) shows that the coefficient on **Dis2014** × **Innovation** is statistically significant, implying that the relationship between proximity to 5A attractions and **CEG** differs across high- and low-innovation cities. The estimates suggest that this relationship is weaker in more innovative cities. Fig 14 provides a complementary visualization of this pattern. Normalizing each group’s predicted CEG to zero at zero distance, the figure shows that residualized CEG declines with distance in both groups, but the decline is less steep in high-innovation cities, consistent with the positive interaction term. One possible interpretation is that firms in more innovative cities rely more on technology-based upgrading and process improvements. Overall, the evidence is consistent with Hypothesis 4.1.

**Table 14:** Urban innovation and environmental governance.

	(1)	(2)	(3)
	Panel A: First stage		Panel B: Second stage (2SLS)
Dependent var	Dis2014	Dis2014 × Innovation	CEG
Dis2014			−7.663***(−4.28)
Dis2014 × Innovation			3.042*(1.70)
Slope	−0.002***(−2.80)	0.000**(2.01)	
Slope×Innovation	−0.001*(−1.61)	−0.004***(−5.67)	
Innovation	0.078***(4.09)	10.552***(687.13)	−31.476*(−1.68)
Controls	Yes	Yes	Yes
Cons	10.096***(16.37)	2.068***(4.81)	71.205***(4.55)
Prefecture effect	Yes	Yes	Yes
SE	Robust	Robust	Robust
N	9097	9097	9097

Note: ***, **, and * denote statistical significance at the 1%, 5%, and 10% levels, respectively. Values in parentheses are t-statistics. Columns with “Robust” report heteroskedasticity-robust standard errors.

#### 5.4.3. Heterogeneity by marketization.

We examine heterogeneity by the degree of city marketization. We define **Market** as an indicator equal to one if a city’s 2014 marketization index is at or above the median across all cities in the sample, and zero otherwise. The marketization index is taken from the Regional Marketization Report (2020). We then estimate Equation (4) by interacting **Dis2014** with **Market**.

**Fig 15:**
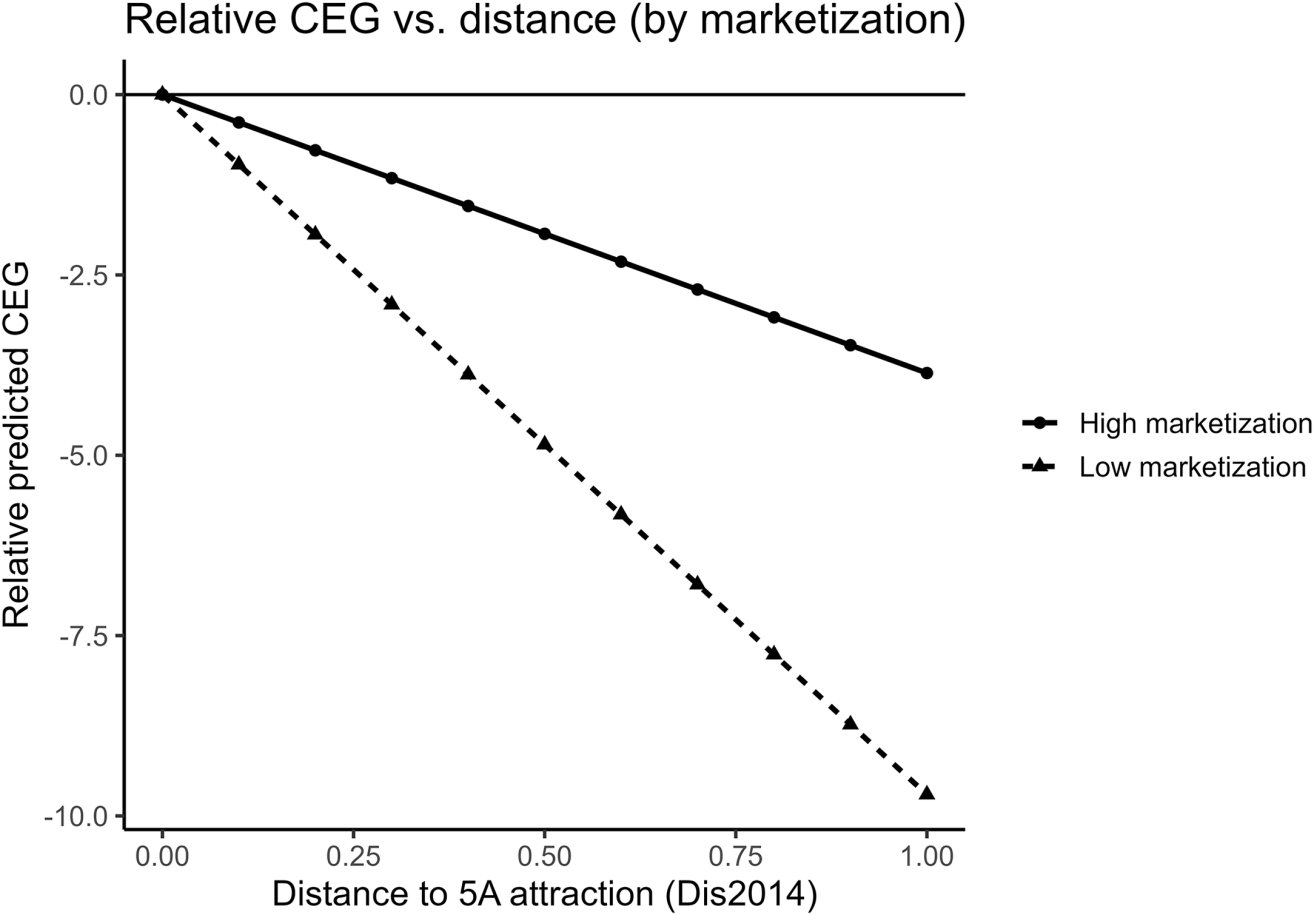
Relative predicted CEG versus distance, by city marketization.

Following Tables 13, 14, we estimate a 2SLS specification that instruments both **Dis2014** and **Dis2014** × **Market** using terrain **slope** and its interaction with **Market**. Table 15 reports a positive and statistically significant coefficient on **Dis2014** × **Market**, implying that the distance-CEG association is weaker in high-marketization cities. Fig 15 visualizes this pattern by plotting relative predicted CEG against distance separately for high- and low-marketization cities, with each group normalized to zero at distance = 0. The high-marketization profile is flatter, consistent with the interaction estimate. One interpretation is that more market-oriented environments improve access to external finance and strengthen market-based incentives, facilitating technology upgrading and cleaner production. Prior studies likewise document that financing constraints can impede firms’ adoption of environmental technologies [66,67]. Overall, the evidence is consistent with Hypothesis 4.2.

**Table 15:** Marketization and environmental governance.

	(1)	(2)	(3)
	Panel A: First stage		Panel B: Second stage (2SLS)
Dependent var	Dis2014	Dis2014 × Market	CEG
Dis2014			−9.704***(−4.04)
Dis2014 × Market			5.844**(2.35)
Slope	−0.001***(−2.70)	0.001**(3.21)	
Slope×Market	−0.002***(−3.24)	−0.004***(−6.21)	
Market	−0.004(−0.20)	10.532***(719.73)	−61.604*(−2.36)
Controls	Yes	Yes	Yes
Cons	10.251***(16.68)	0.268 (0.55)	98.071***(4.00)
Prefecture effect	Yes	Yes	Yes
SE	Robust	Robust	Robust
N	9097	9097	9097

Note: ***, **, and * denote statistical significance at the 1%, 5%, and 10% levels, respectively. Values in parentheses are t-statistics. Columns with “Robust” report heteroskedasticity-robust standard errors.

## 6. Conclusion and limitations

### 6.1 Conclusion

This study examines the relationship between firms’ proximity to nationally designated 5A tourist attractions and corporate environmental governance (CEG) among industrial enterprises. Using 2014 cross-sectional data, we find a robust association in which firms located closer to 5A attractions tend to exhibit stronger CEG outcomes. The auxiliary evidence is consistent with a shared-infrastructure interpretation: proximity is associated with proxies related to traffic accessibility and operating conditions, and firms closer to attractions also tend to display higher fixed-asset intensity, which may reflect greater capacity for compliance-related investment.

Policy implications are therefore conditional. If proximity to major attractions is associated with stronger environmental governance through shared public goods, including transport connectivity and environmental service provision, then policymakers might consider coordinating tourism planning with industrial environmental management. Some aspects of the infrastructure-spillover channel may generalize to other contexts with major tourist destinations or place-based investments. Other aspects are setting-specific, including the 5A designation system and the administrative attention attached to it.

### 6.2 Limitations

Several limitations matter for interpretation. First, the cross-sectional design cannot rule out unobserved confounders, so the estimates should be interpreted as associations rather than definitive causal effects. Second, the IV strategy may violate the exclusion restriction because terrain slope can affect environmental outcomes through drainage, infrastructure costs, and industrial siting, among other channels; IV results are best viewed as a robustness exercise. Third, our CEG measure captures wastewater-treatment intensity and may not reflect broader environmental management. Fourth, missingness in wastewater variables and the composite nature of the cost proxy raise sample-selection and measurement concerns. Future work using panel data, richer environmental outcomes, and alternative settings would help assess robustness and external validity.

## Supporting information

S1 Data (XLSX)
